# Mechanism of hsa_circ_0069443 promoting early pregnancy loss through ALKBH5/FN1 axis in trophoblast cells

**DOI:** 10.1016/j.isci.2024.111608

**Published:** 2024-12-16

**Authors:** Bai-xue Li, Mei-yao Wu, Zhi-hui Wang, Dong-mei Zhou, Jian-qi Li, Bing-feng Lu, Xiao-ling Lin, Yang Zhao, Xiu-jie Sheng

**Affiliations:** 1 Department of Obstetrics and Gynecology, The Third Affiliated Hospital, Guangzhou Medical University, Guangzhou 510150, China; 2 Department of Gynecologic Oncology Research Office, The Third Affiliated Hospital, Guangzhou Medical University, Guangzhou 510150, China; 3 Guangzhou Key Laboratory of Targeted Therapy for Gynecologic Oncology, The Third Affiliated Hospital, Guangzhou Medical University, Guangzhou 510150, China; 4 Guangdong Provincial Key Laboratory of Major Obstetric Diseases, The Third Affiliated Hospital, Guangzhou Medical University, Guangzhou 510150, China; 5 Department of Gynecology, Queen Mary Hospital, Hong Kong, China; 6 Guangdong Provincial Clinical Research Center for Obstetrics and Gynecology, The Third Affiliated Hospital, Guangzhou Medical University, Guangzhou 510150, China; 7 Guangdong-Hong Kong-Macao Greater Bay Area Higher Education Joint Laboratory of Maternal-Fetal Medicine the Third Affiliated Hospital, Guangzhou Medical University, Guangzhou 510150, China

**Keywords:** Pregnancy, Molecular biology, Cell biology

## Abstract

Studies have shown that circRNAs play an important regulatory role in trophoblast function and embryonic development. Based on sequencing and functional experiments, we found that hsa_circ_0069443 can regulate the function of trophoblast cells, and its presence is found in the exosomes secreted by trophoblast cells. It is known that exosomes mediate the interaction between the uterus and embryo, which is crucial for successful pregnancy. We found that trophoblast cell-derived exosomes overexpressing hsa_circ_0069443 promoted the migration and invasion of endometrial stromal cells as well as the EMT process of endometrial glandular epithelial cells, and this process promotes embryo implantation and adhesion, thus proving that a decrease in hsa_circ_0069443 may be the key factor leading to early pregnancy loss. This study also found that hsa_circ_0069443 can bind to the RNA-binding protein demethylase ALKBH5, affecting the overall m6A level of trophoblast cells, and hsa_circ_0069443 and ALKBH5 can regulate the expression level of FN1, verifying the role of the 0069443/ALKBH5/FN1 axis in trophoblast cells and endometrial stromal cells. In summary, this study demonstrates that hsa_circ_0069443 may be a key factor leading to early pregnancy loss, and the regulation of the hsa_circ_0069443/ALKBH5/FN1 axis may provide new insights into early diagnostic markers for early pregnancy loss.

## Highlights

• Hsa_circ_0069443/ALKBH5/FN1 axis affecting the function of trophoblast cells
• Exosomes facilitating the interaction between maternal endometrium and placenta
• Exosomes promote the EMT in endometrial glandular epithelial cells

## Graphical abstract

**Figure:**
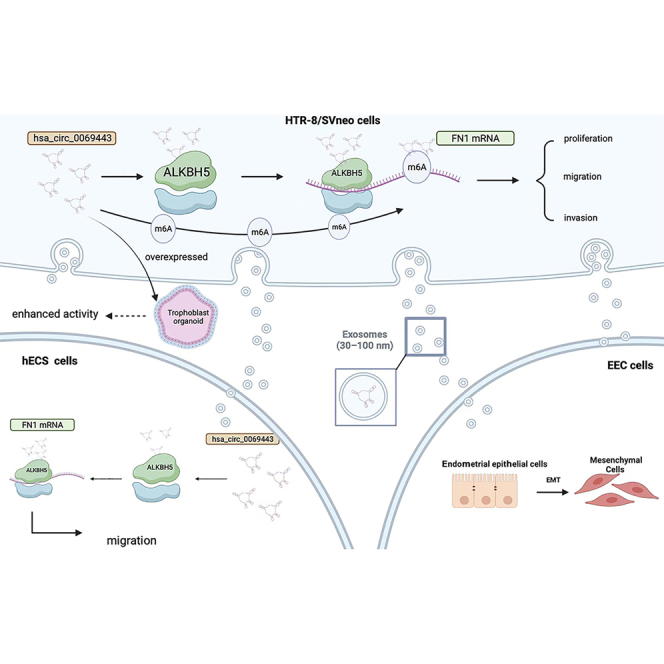

## Introduction

Problems with childbirth have always been a focus of attention in various countries, and early pregnancy loss accounts for about 80% of all pregnancy losses.^1^ It not only affects women’s physical and mental health, but also has an impact on society and families. For such a public health issue, it is important that pregnant women receive the attention of obstetricians and gynecologists. The definition of the number of weeks representing early pregnancy loss is not entirely consistent among different countries. In this article, the definition given by the American College of Obstetricians and Gynecologists (ACOG) is mainly used: intrauterine pregnancy at 12 + 6/7 weeks with no signs of embryo or fetal survival, including empty gestational sac or no fetal heartbeat.^1^ The causes of early pregnancy loss are complex, but mainly include genetic factors, physiological factors, environmental factors, and immune factors.^2^^,^^3^^,^^4^^,^^5^ Among the genetic factors, chromosomal abnormalities (including parental chromosomal abnormalities and embryonic chromosomal abnormalities) account for the majority of early embryo loss (approximately 50%).^6^^,^^7^ Embryonic chromosomal abnormalities are the main cause of early pregnancy loss.^8^ The current mechanism of miscarriage is not fully understood. Studies have shown that non-coding RNAs play important regulatory roles in early pregnancy processes such as embryo implantation, embryo development, and placental formation.^9^ The roles of non-coding RNAs and epigenetics in early pregnancy loss are complex yet crucial. This article aims to further explore the role of non-coding RNAs in early pregnancy loss, which can help reveal the mechanisms underlying early pregnancy loss and provide theoretical basis for its prevention and treatment.

Noncoding RNAs (ncRNAs) play important biological functions at the RNA level, and can regulate important life activities by participating in chromosome remodeling, gene transcription, and post transcriptional modifications.^10^ NcRNAs include circular RNAs (circRNAs), long noncoding RNAs (lncRNAs), microRNAs (miRNAs), small nuclear RNAs (snRNAs), and so on. Among them, circRNAs are formed by reverse splicing to connect the 3′ end of the exon connected to its own 5′ end or to upstream exon through 3′, 5′-phosphate diester bonds, forming a closed structure with reverse splicing connection sites.^11^^,^^12^ This structure makes it more stable with a longer half-life.^13^ With the improvement of high-throughput RNA sequencing and computational methods, the vast majority of circRNAs have been identified. Studies have found that over 10,000 circRNAs are expressed in pre-transplant human embryos, and most of them exhibit developmental stage specific expression.^11^^,^^14^^,^^15^^,^^16^^,^^17^^,^^18^ Li et al.^19^ found that in patients with early recurrent spontaneous abortion (RSA), 123 differentially expressed circRNAs were found compared to those in women with normal pregnancies, including 78 upregulated and 45 downregulated circRNAs. Another study demonstrated the molecular mechanism by which the circ-ZUFSP regulates trophoblast migration and invasion through an *in vitro* study of the effect of circ-ZUFSP on trophoblast function and provided new indicators for the diagnosis and treatment of RSA.^20^ There is relatively little research on circRNAs in the field of abortion; therefore, this article mainly explores the mechanism and regulation of circRNAs in early pregnancy loss.

We performed high-throughput sequencing of RNA in the placental villi of four pairs of patients with early pregnancy loss and patients with normal healthy early pregnancy. At the same time, we collected the villi of 95 patients with early pregnancy loss and 132 patients with induced abortion for quantitative real-time reverse transcription PCR (qRT-PCR) analysis and screened out the differentially expressed hsa_circ_0069443. Currently, there are no reports on this molecule domestically or internationally. We aimed to study the potential functions and mechanisms of hsa_circ_0069443 in patients with early pregnancy loss, with the hopes of it becoming a new target for the treatment of early pregnancy loss.

## Results

### Hsa_circ_0069443 is downregulated in patients with early pregnancy loss

**Figure 1:**
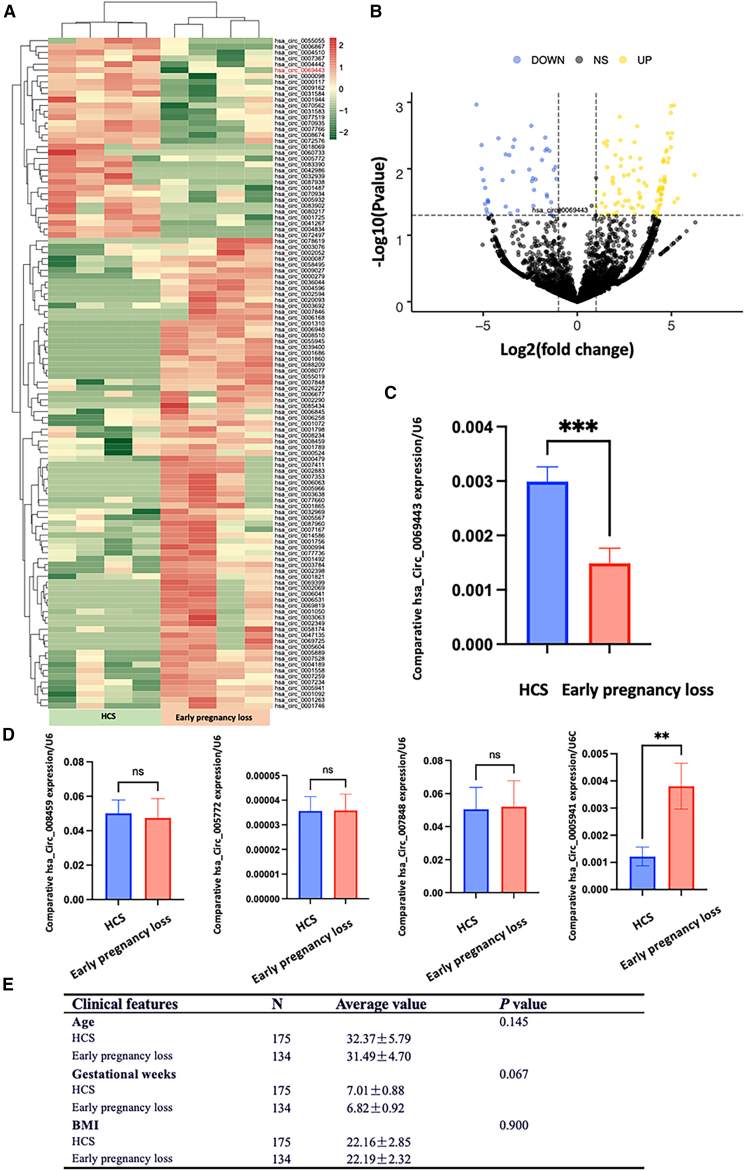
Differential expression circRNAs in spontaneous abortion and normal pregnancy (A) Hierarchical clustering analysis was performed on upregulated and downregulated circRNA expression in placental villi of four pairs of patients with normal pregnancy and early pregnancy loss. Red indicates high expression and green indicates low expression. (B) Differential expression of circRNAs in patients with early pregnancy loss and normal pregnancy. (C) Detection of hsa_circ_0069443 in placental villous tissue from 175 patients with early pregnancy loss and 134 normal control patients using qRT-PCR. The level of hsa_circ_0069443 in the early pregnancy group was higher than that in the control group (∗∗∗*p* < 0.001). (D) hsa_circ_008459 showed no statistically significant difference in expression between 11 pairs of early pregnancy patients and patients with early pregnancy loss in chorionic villi tissues (*p* > 0.05). Similarly, hsa_circ_005772 exhibited no statistically significant difference in expression between 12 pairs of early pregnancy patients and patients with early pregnancy loss in chorionic villi tissues (*p* > 0.05). The expression of hsa_circ_007848 also showed no statistically significant difference between 12 early pregnancy cases and 11 cases of early pregnancy loss in chorionic villi tissues (*p* > 0.05). In a cohort of 48 early pregnancy patients and 46 patients with early pregnancy loss, the expression level of hsa_circ_0005941 was significantly lower in the early pregnancy group compared to the control group (*p* < 0.05). (E) There were no significant differences in age, gestational age, and BMI between the early pregnancy loss group and the early pregnancy group. Data are expressed as the mean ± SEM. ns, *p* ≥ 0.05; ∗, *p* < 0.05; ∗∗, *p* < 0.01; ∗∗∗, *p* < 0.001; ∗∗∗∗∗, *p* < 0.0001. SEM, standard error of the mean.

We performed RNA high-throughput sequencing in four patients with early pregnancy loss and four patients with normal early pregnancy, and used a *p*-value <0.05 and | log2FoldChange |>1 as the criteria for defining differentially expressed circRNAs. According to the sequencing results, 95 upregulated circRNAs and 43 downregulated circRNAs were identified (Figures 1A and 1B). Based on the high-throughput sequencing results of chorionic villi from 4 patients with early pregnancy loss and healthy early pregnant controls, we identified 5 significantly different indicators. We then gradually increased the sample size of tissue samples to further validate the differential expression of these indicators. According to the validation results from tissue samples, hsa_circ_008459, hsa_circ_005772, and hsa_circ_007848 showed no significant differences between the chorionic villi tissues of normal healthy early pregnant controls and patients with early pregnancy loss. Hsa_circ_0005941 was found to be downregulated in the early pregnancy group compared to the control group. We have conducted further mechanistic studies and are currently preparing the research results for publication. When the sample size was expanded to 175 cases of early pregnancy patients and 134 cases of early pregnancy loss patients, the expression level of hsa_circ_0069443 in early pregnancy patients remained significantly higher than in the control group (Figures 1C and 1D). We have supplemented the clinical characteristics of these pregnant women and included the additional information in Table S5. We compared the age, gestational age, and BMI of these pregnant women using SPSS software and found no significant differences between the early pregnancy group and the early pregnancy loss group (Figure 1E).

### Hsa_circ_0069443 promotes the proliferation, migration, and invasion of trophoblast cells

**Figure 2:**
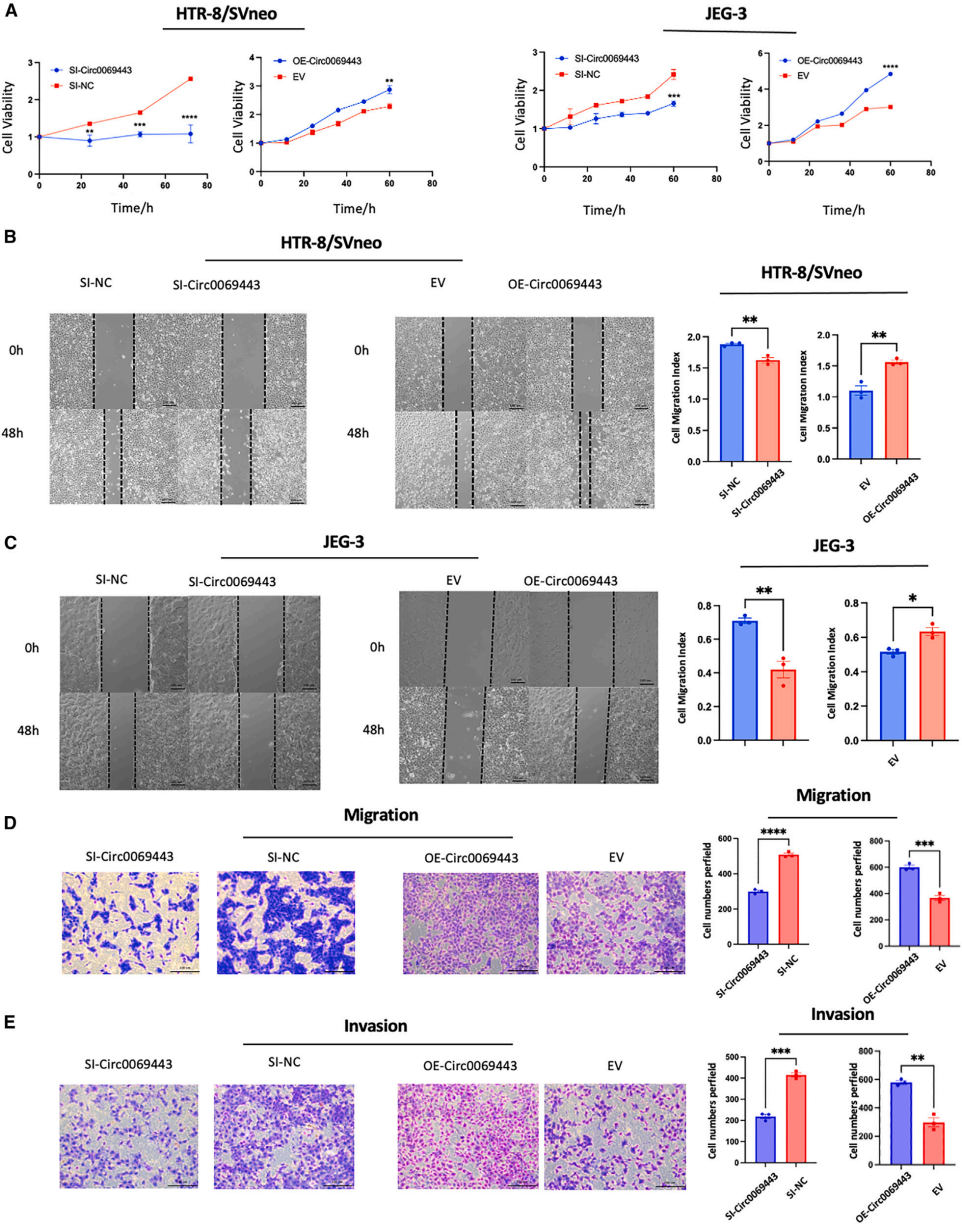
The effect of hsa_circ_0069443 on the proliferation, migration, and invasion ability of trophoblasts (A) HTR-8/SVneo and JEG-3 cells were transfected with siRNA and overexpression plasmids. Knockdown of hsa_circ_0069443 significantly reduced cell proliferation while overexpressing hsa_circ_0069443 promote the proliferation of trophoblasts. (B and C) In HTR-8/SVneo and JEG-3 cells, knockdown of hsa_circ_0069443 inhibited trophoblastic cell migration and overexpression of hsa_circ_0069443 promoted trophoblast cell migration. Images were captured at a magnification of 40×. (D and E) In HTR-8/SVneo cells, knockdown of hsa_circ_0069443 inhibits the migration and invasive ability of trophoblasts, whereas overexpression of hsa_circ_0069443 promotes the migration and invasive ability of trophoblasts. The cells were stabilized with 0.5% crystal violet and images were captured at a magnification of 100×. Scale bar = 100 μm in (B,C). scale bar = 200 μm in others; The results shown are representative of three separate experiments, *n* = 3; Data are expressed as the mean ± SEM. ns, *p* ≥ 0.05; ∗, *p* < 0.05; ∗ ∗, *p* < 0.01; ∗ ∗ ∗, *p* < 0.001; ∗∗∗∗∗, *p* < 0.0001. NC, negative control; Si-hsa_circ_0069443, hsa_circ_0069443 siRNA; EV, empty vector; OE hsa_circ_0069443, overexpressing hsa_circ_0069443; SEM, standard error of the mean.

**Figure 3:**
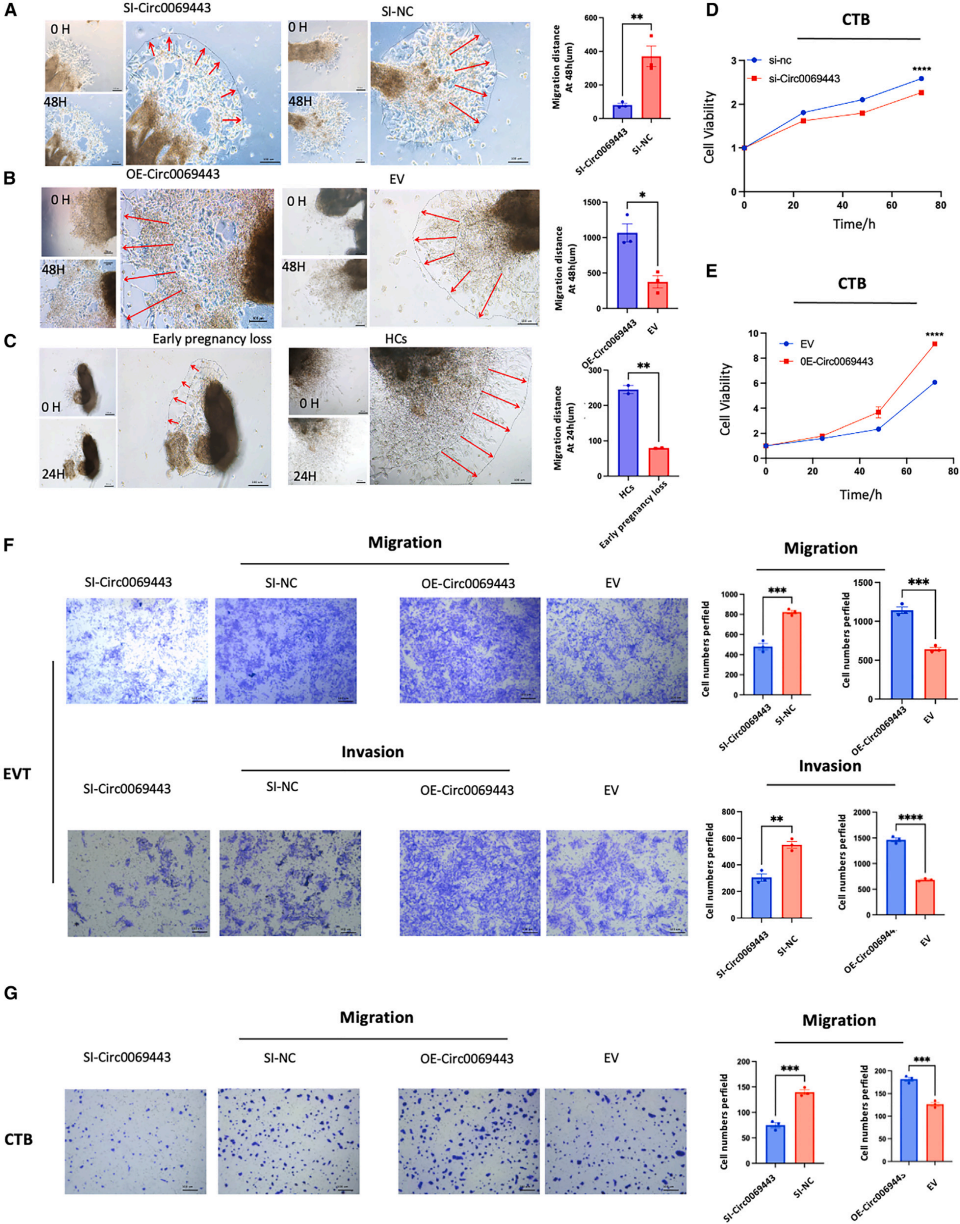
Growth of trophoblasts in villous tissue explant culture (A and B) Knockdown of hsa_circ_0069443 reduced the migration distance of villous trophoblast cells in explants, whereas overexpressing hsa_circ_0069443 increased the migration distance of villous trophoblasts in explants. (C) Compared to villi in healthy early pregnancy patients, the migration ability of villous trophoblasts was relatively poor in patients with early pregnancy loss. Images were captured at a magnification of 100×. (D and E) CTB cells were transfected with siRNA and overexpression plasmids. Knockdown of hsa_circ_0069443 significantly reduced cell proliferation while overexpressing hsa_circ_0069443 promote the proliferation of trophoblasts. (F) In EVT cells, knockdown of hsa_circ_0069443 inhibits the migration and invasive ability of trophoblasts, whereas overexpression of hsa_circ_0069443 promotes the migration and invasive ability of trophoblasts. The cells were stabilized with 0.5% crystal violet and images were captured at a magnification of 40×. (G) In CTB cells, knockdown of hsa_circ_0069443 inhibits the migration ability of trophoblasts, whereas overexpression of hsa_circ_0069443 promotes the migration ability of trophoblasts. The cells were stabilized with 0.5% crystal violet and images were captured at a magnification of 40×. Scale bar = 100 μm in (A,B,C,F,G); The results shown are representative of three separate experiments, *n* = 3; Data are expressed as the mean ± SEM. ns, *p* ≥ 0.05; ∗, *p* < 0.05; ∗∗, *p* < 0.01; ∗∗∗, *p* < 0.001; ∗∗∗∗∗, *p* < 0.0001. NC, negative control; Si-hsa_circ_0069443, hsa_circ_0069443 siRNA; EV, empty vector; OE hsa_circ_0069443, overexpressing hsa_circ_0069443; SEM, standard error of the mean.

To study the biological role of hsa_circ_0069443 in patients with early pregnancy loss we knocked down or overexpressed hsa_circ_0069443. Transfection of small interfering RNA (siRNA) and overexpression plasmids into HTR-8/SVneo and JEG-3 cells showed that knockdown of hsa_circ_0069443 significantly reduced cell proliferation, while overexpressing hsa_circ_0069443 promoted the proliferation of trophoblasts according to Cell Counting kit 8 (CCK-8) proliferation assays (Figure 2A). Wound healing experiments showed that overexpression of hsa_circ_0069443 enhanced cell migration, while knocking down hsa_circ_0069443 inhibited migration (Figures 2B and 2C). In addition, Transwell assays were used to study the significance of hsa_circ_0069443 in cell migration (Figures 2D and 2E). Overexpression of hsa_circ_0069443 increased cell migration, while knockdown hsa_circ_0069443 decreased cell migration compared with that in the control group. Transwell invasion experiments showed that compared with that in the empty vector (EV) group, overexpression of hsa_circ_0069443 increase cell invasion, while hsa_circ_0069443 knockdown decreased cell invasion. Further confirmation of the effect of hsa_circ_0069443 on trophoblastic cell migration was based on the explant culture model of early pregnancy villi, to better simulate the differentiation process of trophoblasts in the early development of the placenta. After 48 h of cultivation, spindle shaped trophoblasts could be seen migrating from the tip of the villi. Reference the transfection method of HTR-8/SVneo cells to knockdown or overexpress hsa_circ_0069443, strong red fluorescence was observed at 24 h after Cy3-siRNA transfection into villous explants (Figure S1C), indicating that Lipofectamine 3000 can effectively transfect exogenous siRNA into villous explants. Compared with that in the control group, cy3-si-hsa_circ_0069443 reduced the migration distance of villous trophoblast cell migration in the explants, whereas overexpression of hsa_circ_0069443 increased the migration distance of villous trophoblasts in the explants (Figure 3 A, B). Compared with the villi in patients with healthy early pregnancy, the villous trophoblast cell migration in patients with early pregnancy loss was relatively poor (Figure 3C). Based on the above experimental results, we deduced that hsa_circ_0069443 can promote the proliferation, migration, and invasion of trophoblast cells.

We conducted embedding and fixation of early pregnancy chorionic villi tissues and employed immunohistochemical techniques for detection. Cytokeratin 7 (CK7) was employed as a marker for trophoblast cells, and a green fluorescence-labeled hsa_circ_0069443 probe was used. CK7 staining and fluorescence *in situ* hybridization were performed on tissue sections. Based on the results from fluorescence *in situ* hybridization and immunohistochemistry, we found that hsa_circ_0069443 is present in cytotrophoblasts (CTB), syncytiotrophoblasts (STBs), and extravillous trophoblasts (EVTs), with a greater abundance in CTB and EVT (Figure S1A). We isolated and cultured primary cytotrophoblast (CTB) and EVT cells from early pregnancy chorionic villi to conduct cell functional experiments within our time and resource limitations. SiRNA and overexpressing plasmids were transfected into CTB and EVT cells. Cell proliferation experiments using CCK-8 assay showed that knockdown of hsa_circ_0069443 significantly reduced CTB cell proliferation, while overexpression of hsa_circ_0069443 promoted CTB cell proliferation (Figure 3D and 3E). Additionally, the significance of hsa_circ_0069443 in the migration of CTB and EVT cells was studied using Transwell assay. The overexpression group showed increased migration to the bottom chamber compared to the control group, whereas the knockdown group exhibited reduced cell migration compared to the control group(Figures 3F and 3G). Transwell invasion assay demonstrated that in EVT cells, the overexpression group had more cells invading the basement membrane gel compared to the empty vector (EV) group, while the knockdown group had fewer invaded cells compared to the control group(Figure 3F).

### Hsa_circ_0069443 functions in conjunction with ALKBH5

**Figure 4:**
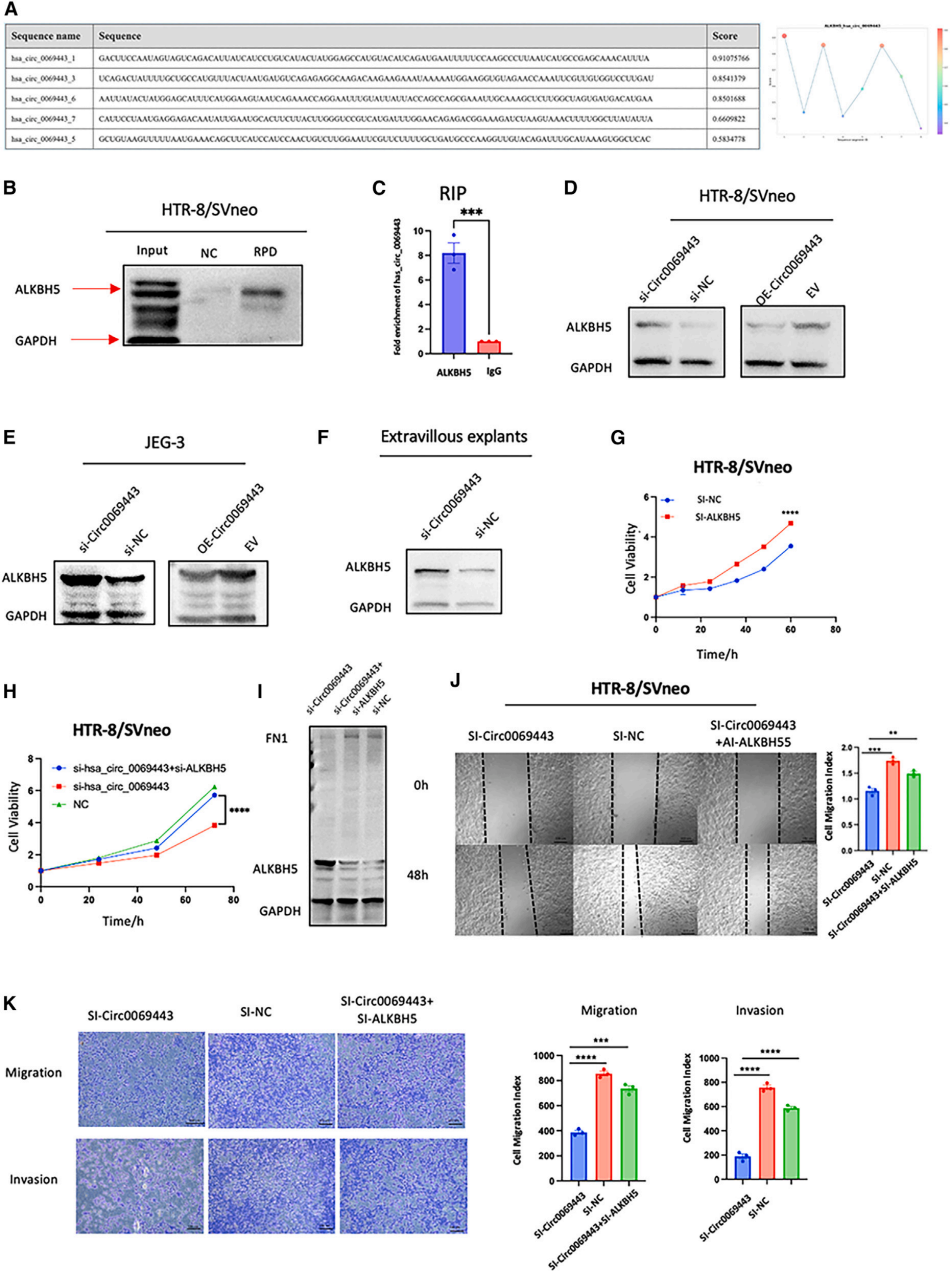
ALKBH5 as an RBP of hsa_circ_0069443 (A) According to RBPSuite, a bioinformatics website for predicting RBPs), ALKBH5 binding sites and scores were determined. (B) Immunoblot analysis of ALKBH5 after RNA-pulldown assay showing its specific association with has_circ_0069443. (C) RIP assays showing higher levels of hsa_circ_0069443 in the ALKBH5 antibody precipitated complex compared to the IgG group. (D–F) In the HTR-8/SVneo and JEG-3 cells, knockdown of hsa_circ_0069443 enhanced the level of ALKBH5 protein, while conversely, overexpressing hsa_circ_0069443 decreased the protein of level of ALKBH5, and the same results were obtained in the villous tissue of the explant. (G) In the HTR-8/SVneo cells, knockdown of ALKBH5 significantly promote the proliferation of trophoblasts. (H) Knockdown of ALKBH5 partially reversed the effect of si-hsa_circ_0069443 HTR-8/SVneo cell proliferation. (I) The protein expression of FN1 and ALKBH5 when HTR-8/SVneo cells were knocked down has_circ_0069443, or knocked down has_circ_0069443 and knocked down ALKBH5, or control. (J and K) Knocked down ALKBH5 can partially reverse si hsa_circ_0069443’s effect on HTR-8/SVneo cell migration and invasion. Images were captured at a magnification of 40×. Scale bar = 100 μm in (J,K). The results shown are representative of three separate experiments, *n* = 3; Data are expressed as the mean ± SEM. ns, *p* ≥ 0.05; ∗, *p* < 0.05; ∗∗, *p* < 0.01; ∗∗∗, *p* < 0.001; ∗∗∗∗∗, *p* < 0.0001. NC, negative control; Si-hsa_circ_0069443, hsa_circ_0069443 siRNA; EV, empty vector; OE hsa_circ_0069443, overexpressing hsa_circ_0069443; Si-has_circ_0069443+Si-ALKBH5, hsa_circ_0069443 siRNA and ALKBH5 siRNA; SEM, standard error of the mean.

As predicted by bioinformatics software (RBPsuite; http://www.csbio.sjtu.edu.cn/bioinf/RBPsuite/), the RNA binding protein (RBP) AlkB homolog 5, RNA demethylase (ALKBH5) might be associated with hsa_circ_0069443(Figure 4A). To test this hypothesis, RNA pull-down experiments and RNA immunoprecipitation (RIP) assays were performed on the HTR-8/SVneo cell line, the results showed that ALKBH5 could be hsa_circ_0069443 probe successfully pulled down (Figure 4B), and higher levels of hsa_circ_0069443 in the ALKBH5 antibody precipitated complex compared to the IgG group (Figure 4C). We used Western blotting to show that knocking down has_circ_0069443 in HTR-8/SVneo and JEG-3 cell lines increased ALKBH5 protein levels compared to the control group, while overexpressing hsa_circ_0069443 led to a decrease in ALKBH5 protein(Figures 4D and 4E).The same results were obtained in the villous tissue of the explant (Figure 4F). Previous literature has reported that ALKBH5 inhibits the migration and invasion of trophoblast cells using *in vitro* cell experiments.^21^ In addition, CCK-8 cell proliferation experiments in the HTR-8/SVneo cell line showed that knocking down *ALKBH5* promoted trophoblast cell proliferation (Figure 4G). To further confirm the relationship between the two, we conducted salvage experiments in HTR-8/SVneo cells. Knockdown of hsa_circ_0069443 could reverse the inhibitory effect on cell proliferation, migration and invasion functions produced by knocking down *ALKBH5* expression (Figures 4H–4K). The above data showed that hsa_circ_0069443 can directly interact with ALKBH5 protein, regulate the protein level of ALKBH5, and affects the proliferation, migration, and invasion abilities of trophoblast cells.

### The relationship between hsa_circ_0069443 and ALKBH5 and *FN1* mRNA

**Figure 5:**
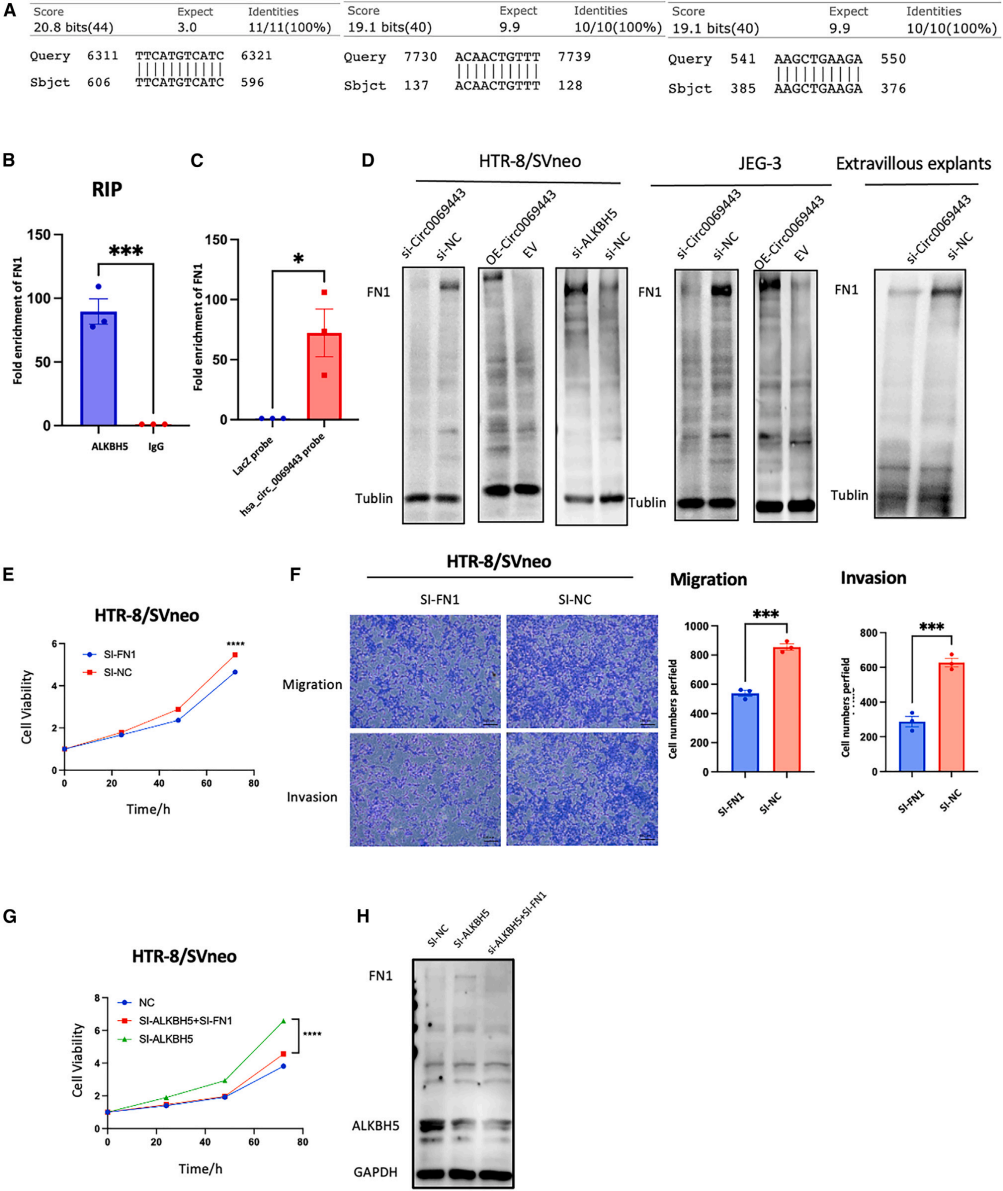
The relationship between hsa_circ_0069443 and ALKBH5 and FN1 mRNA (A) hsa_circ_0069443 has base complementary pairing with the FN1mRNA sequence. (B) RIP assays confirmed that ALKBH5 was connected with FN1mRNA. (C) RNA pull-down experiment proves that hsa_circ_0069443 is connected to FN1. (D) In HTR-8/SVneo and JEG-3 cells, knockdown or overexpression of hsa_circ_0069443 can affect the protein expression of FN1, and knockdown of hsa_circ_0069443 in villous explants also reduced the expression of FN1 protein. (E) HTR-8/SVneo cells were transfected with siRNA. Knockdown of FN1 significantly reduced cell proliferation. (F) In HTR-8/SVneo cells, knockdown of FN1 inhibits the migration and invasive ability of trophoblasts. The cells were stabilized with 0.5% crystal violet and images were captured at a magnification of 40×. Scale bar = 100 μm. (G) Knockdown FN1 partially reversed the effect of si-ALKBH5 on HTR-8/SVneo cell proliferation. (H) The protein expression of FN1 and ALKBH5 when HTR-8/SVneo cells were knockdown ALKBH5, or knockdown ALKBH5 and knockdown of FN1, or control. The results shown are representative of three separate experiments, *n* = 3. Data are expressed as the mean ± SEM. ns, *p* ≥ 0.05; ∗, *p* < 0.05; ∗∗, *p* < 0.01; ∗∗∗, *p* < 0.001; ∗∗∗∗∗, *p* < 0.0001. SEM, standard error of the mean.

ALKBH5 is an m6A demethylase, and m6A is the most common internal modification of mRNA.^22^ To clarify the downstream regulatory mechanism of ALKBH5, RNA immunoprecipitation sequencing (RIP-seq) was performed by pulling down ALKBH5 in the JEG-3 cell line (Table S6). According to the sequencing results, using IP/input ≥2, FPKM (Fragments Per Kilobase per Million mapped fragments)≥ 1, and *p* < 0.05 as RIP thresholds, and a total of 3304 genes were found to have abnormal expression. According to the SRAMP website (http://www.cuilab.cn/sramp), predicting whether downstream molecules have N6-methyladenosine methylation sites and screening hsa_circ_0069443 based on NCBI (https://www.ncbi.nlm.nih.gov/)blast function, we found that the hsa_circ_0069443 sequence and the *FN1* mRNA sequence (encoding fibronectin 1) have multiple complementary base pairing (Figure 5A). The connection between ALKBH5 and *FN1* was confirmed through RIP experiments(Figure 5B), and hsa_circ_0069443 was demonstrated through RNA pull-down experiments to be connected to *FN1* (Figure 5C). Knocking down *ALKBH5* in the HTR-8/SVneo cell line increased the protein level of FN1. In the HTR-8/SVneo cell lines、JEG-3 cell lines and extravillous explants, knocking down or overexpressing hsa_circ_0069443 also influence the levels of the FN1 protein (Figure 5D). Transfection of small interfering RNA (siRNA) plasmids into HTR-8/SVneo cells showed that knockdown of hsa_circ_0069443 significantly reduced cell proliferation according to Cell Counting kit 8 (CCK-8) proliferation assays(Figure 5E). Transwell assays were used to study the significance of FN1 in cell migration and invasion, knockdown hsa_circ_0069443 decreased cell migration and cell invasion compared with that in the control group(Figure 5F). CCK-8 cell proliferation experiments showed that knocking down of ALKNH5 after knocking down *FN1* could reverse cell proliferation (Figures 5G and 5H). Finally, it was determined that *FN1* was the target of ALKBH5.

The above results indicate that ALKBH5 targets *FN1* mRNA, and hsa_circ_0069443 might interact with *FN1* mRNA via complementary base pairing.

### The expression level of hsa_circ_0069443 affects the m6A level of total RNA in trophoblast cells

**Figure 6:**
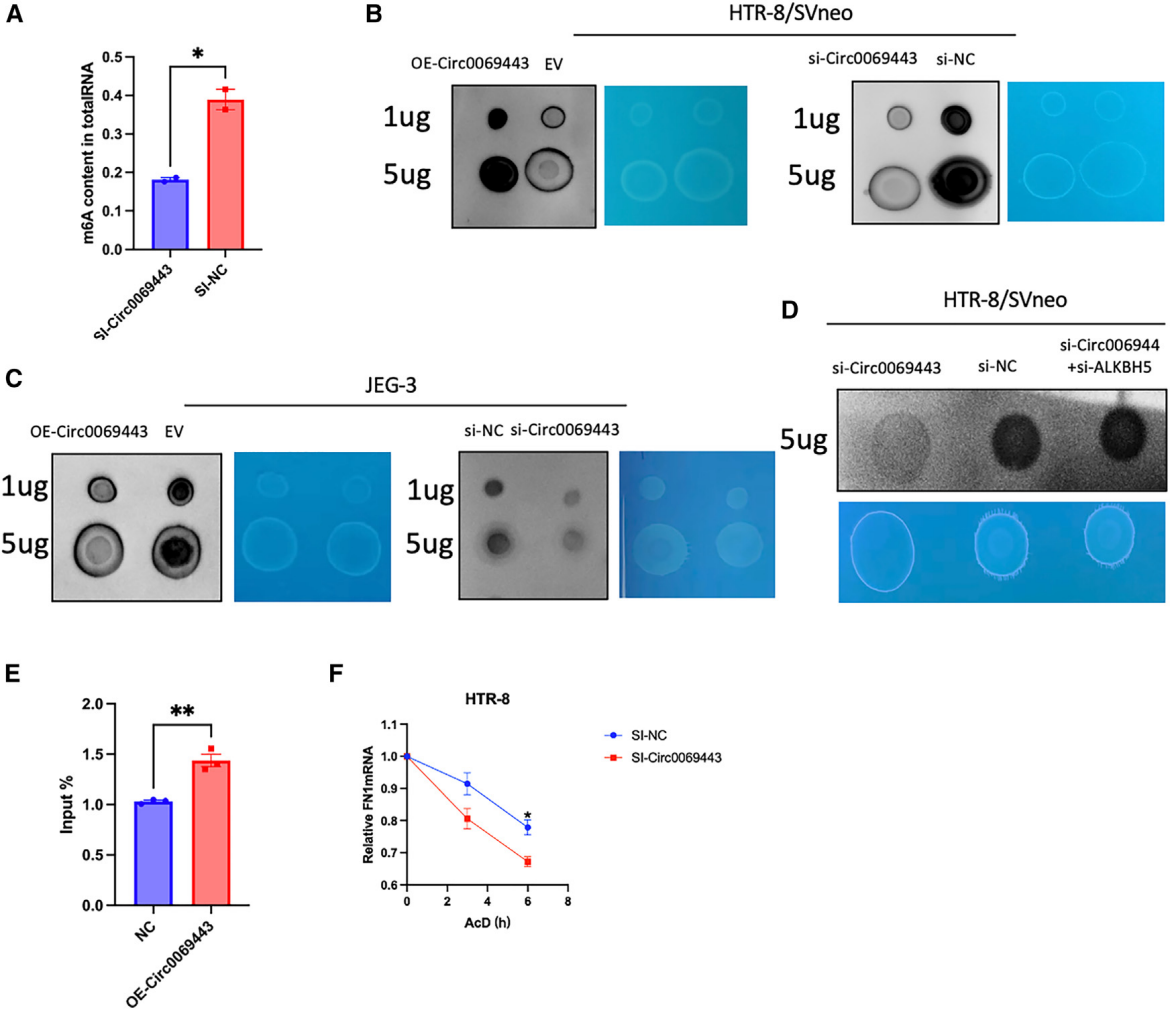
The expression level of hsa_circ_0069443 affects the m6A level of total RNA in trophoblast cells (A) Colorimetry assays in the HTR-8/SVneo cells shown that knockdown of hsa_circ_0069443 reduced the overall m6A level. (B and C) Dot blot assays In the HTR-8/SVneo cells and JEG-3 cells showed that compared with the negative control group, hsa_circ_0069443 knockout reduced the total m6A levels of trophoblasts and overexpression of hsa_circ_0069443 increased the total m6A level. (D) Knockdown of ALKBH5 partially reversed the effect of hsa_circ_0069443 knockdown on methylation levels. (E) Overexpression of hsa_circ_0069443 was detected by meRIP-qRT-PCR. The m6A level in FN1 mRNA was higher than that in the control group. (F) Knockdown of hsa_circ_0069443 reduces the stability of *FN1* mRNA. The results shown are representative of three separate experiments, *n* = 3. Data are expressed as the mean ± SEM. ns, *p* ≥ 0.05; ∗, *p* < 0.05; ∗∗, *p* < 0.01; ∗∗∗, *p* < 0.001; ∗∗∗∗∗, *p* < 0.0001. SEM, standard error of the mean.

We have proven that the relationship between hsa_circ_0069443, ALKBH5 protein, and *FN1* mRNA ultimately leads to changes in trophoblast cell function by affecting the expression of *FN1*. Next, whether ALKBH5, as an m6A demethylase, changes the total RNA m6A methylation level of trophoblasts was first confirmed by colorimetry in the HTR-8/SVneo cell line. Knockdown of hsa_circ_0069443 reduced the overall m6A level (Figure 6A). Further analysis of hsa_circ_0069443 in HTR-8/SVneo and JEG-3 cell lines through dot blot experiments were used to compare the effects of knockdown, overexpression, and overall m6A levels with the negative control group. The results confirmed that knockdown of hsa_circ_0069443 downregulated the total m6A level of trophoblasts compared with that in the negative control group, and overexpression of hsa_circ_0069443 increased the total m6A level. Under conditions of knockdown of hsa_circ_0069443, further knockdown of *ALKBH5* restored the methylation level(Figures 6B–6D). To determine the function of m6A modified *FN1* mRNA, the SRAMP online tool was used to predict the modification site, and a Rach m6A sequence was found in the 3′ UTR region of *FN1* (Table S1). Methylation-related RNA immunoprecipitation (MeRIP)-qRT-PCR showed that overexpression of hsa_circ_0069443 induced an increase in the m6A levels of *FN1* mRNA compared with that in the control group (Figure 6E). We further found that knockdown of hsa_circ_0069443 reduce the mRNA stability of *FN1* (Figure 6F), resulting in a decrease in FN1protein levels. The results indicated that overexpression of hsa_circ_0069443 upregulated the methylation level of *FN1* mRNA, thereby increasing its stability.

### Hsa_circ_0069443 exists in exosomes derived from trophoblast cells and promotes endometrial-mesenchyme transition (EMT) and migration

**Figure 7:**
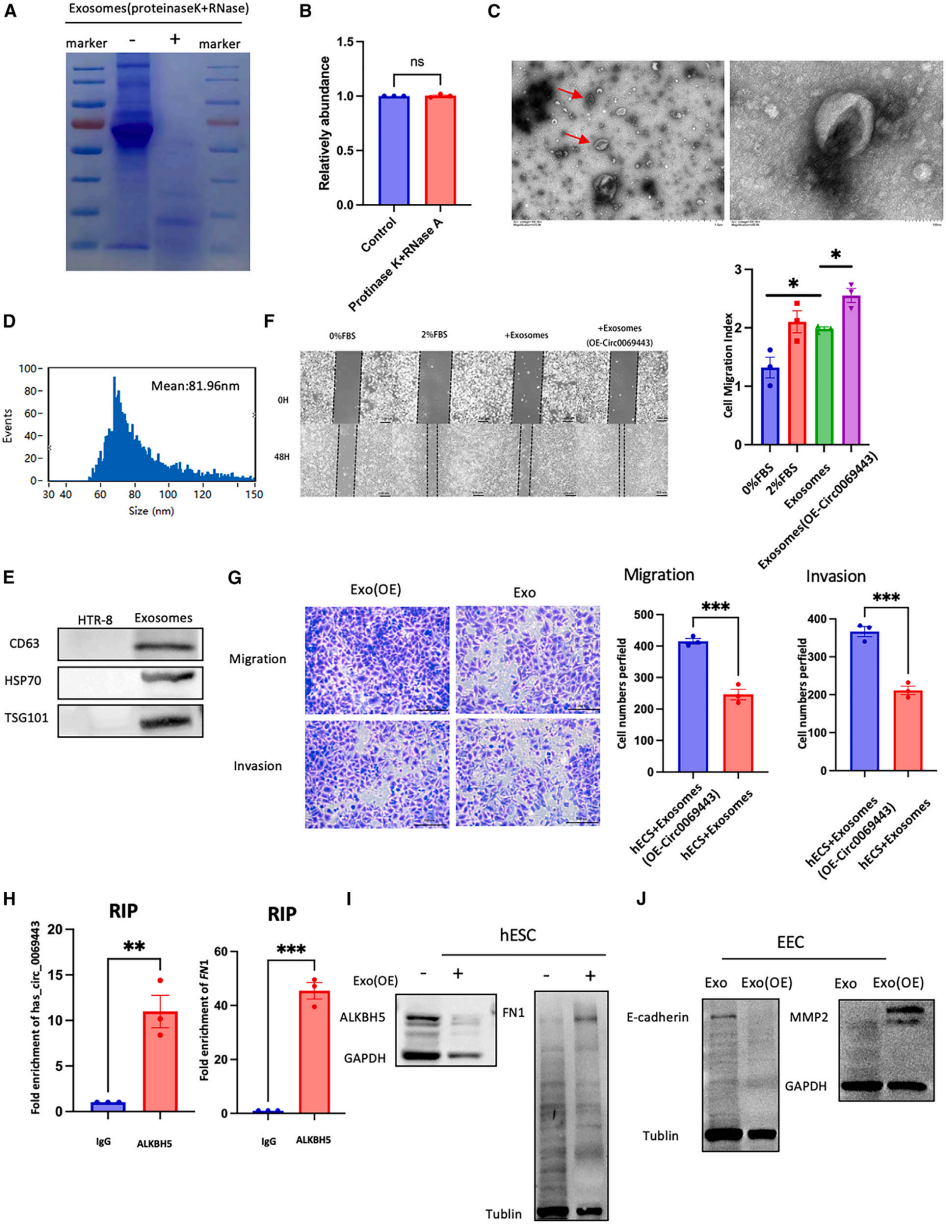
Hsa_circ_0069443 exists in exosomes derived from trophoblast cells and promotes endometrial EMT and migration (A) Proteins in exosomes are largely degraded by proteinase K. (B) qRT-PCR results indicating that hsa_circ_0069443 in exosomes cannot be degraded by proteinase K and RNase A. (C) Electron micrographs showing that the vesicles are circular or disc-shaped. (D) Nanoparticle tracking analysis (NTA) showing that the vesicles had an average size of 81.96 nm. (E) Western blotting analysis confirmed the levels of CD63, TSG101, and HSP70 (extracellular protein markers). (F) A wound healing assay showing that exosomes from cells overexpressing hsa_circ_0069443 significantly enhanced the migration ability of cells. Images were captured at a magnification of 40×. Scale bar = 100 μm. (G) Transwell experiments indicate that the addition of exosomes from cells overexpressing hsa_circ_0069443 can promote the migration and invasion. Images were captured at a magnification of 100×. Scale bar = 200 μm. (H) RIP experiments showing that in hESC cells, hsa_circ_0069443 and FN1 mRNA can be linked to ALKBH5. (I) Compared with hESC alone, the addition of exosomes from cells overexpressing hsa_circ_0069443 resulted in changes in ALKBH5 and FN1 protein levels. (J) After adding exosomes that overexpressed hsa_circ_0069443, the levels of E-cadherin were significantly reduced, while the levels of matrix metalloproteinase (MMP2) were significantly upregulated. The results shown are representative of three separate experiments, *n* = 3. Data are expressed as the mean ± SEM. ns, *p* ≥ 0.05; ∗, *p* < 0.05; ∗ ∗, *p* < 0.01; ∗ ∗ ∗, *p* < 0.001; ∗∗∗∗∗, *p* < 0.0001. SEM, standard error of the mean.

We extracted exosomes from the supernatant of HTR-8/SVneo cells using a polyethylene glycol (PEG) reagent kit method and high-speed centrifugation. The extracted extracellular vesicles were evenly divided into two parts, with one part added with proteinase K and RNase A for degradation analysis. The other parts of the exosomes were used for qRT-PCR detection to confirm the presence of hsa_circ_0069443. Proteins extracted from the exosomes were stained with Coomassie blue, and the results confirmed that the proteins in the exosome-rich components were largely degraded by proteinase K (Figure 7A). The qRT-PCR results indicated that hsa_circ_0069443 in the exosomes could not be degraded by proteinase K and RNase A (Figure 7B), indicating that hsa_circ_0069443 is effectively protected by the extracellular membrane and its circular structure. Electron microscopy showed that the vesicles were circular or disc-shaped (Figure 7C). Nanoparticle tracking analysis (NTA) showed an average size of 81.96 nm (Figure 7D). Western blotting analysis confirmed the presence of CD63, tumor susceptibility 101 (TSG101), and heat shock protein 70 (HSP70) (extracellular protein markers) (Figure 7E). We further isolated and cultured primary trophoblast cells from 9 pairs of early pregnancy loss and normal pregnancy chorionic villi tissues, respectively. We extracted and isolated exosomes from cell culture supernatants, followed by RNA extraction, and conducted qRT-PCR analysis. The results revealed a significantly decreased expression level of hsa_circ_0069443 in the exosomes derived from trophoblast cells of patients with early pregnancy loss (Figure S1B).

Research has shown that during embryo implantation, the activity of endometrial stromal cells increases and moves away from the implantation site, allowing trophoblasts to invade the endometrial stroma.^23^ To further confirm the effect of exosomes on the endometrium, exosomes isolated from HTR-8/SVneo cells were added to the normal endometrial stromal cells (hESC). The wound healing test showed that endometrial cell migration did not change in the presence of serum-free medium. Cells treated with trophoblast exosomes showed a decrease in cell migration ability compared to that of the 2% serum control. After overexpression of hsa_circ_0069443 in trophoblast cells, exosomes were extracted and added to hESC cells. The wound healing test showed that exosomes from cells overexpressing hsa_circ_0069443 significantly enhanced the migration ability of cells (Figure 7F). Transwell experiments indicated that the addition of exosomes promoted the migration and invasion of hESC cells (Figure 7G). Next, we sought to determine whether the molecular mechanism of endometrial stromal cells is consistent with that of trophoblasts. We carried out RIP analysis in hESC cells and found higher levels of hsa_circ_0069443 and *FN1* mRNA in the ALKBH5 antibody precipitated complex than in the IgG group (Figure 7H). After co-incubation of hESC cells and trophoblast cell-derived exosomes overexpressing hsa_circ_0069443 for 48 h, the total proteins were extracted. Compared with hESC alone, the addition of exosomes resulted in changes in ALKBH5 and FN1 protein levels (Figure 7I).

Previous studies have suggested that the EMT of endometrial cells during the implantation window can promote the implantation process.^24^ Therefore, we further investigated the influence of trophoblast-derived exosomes on the epithelial-mesenchymal transition (EMT) of endometrial epithelial cells (EECs). Therefore, we further explored the influence of exosomes derived from trophoblast cells on the epithelial-mesenchymal transition (EMT) of endometrial epithelial cells (EECs). After incubating the exosomes with EECs for 48 h, the total proteins were extracted, and the EMT-related proteins were detected by western blotting. After adding exosomes that overexpressed hsa_circ_0069443, the levels of E-cadherin, an epithelial marker, in EECs were significantly reduced, while the levels of matrix metalloproteinase (MMP2), a stromal marker, were significantly upregulated (Figure 7J). These results indicate that hsa_circ_0069443 is secreted through exosomes and promotes EMT of EEC_S_.

### Hsa_circ_0069443 promotes trophoblast organoids proliferation

**Figure 8:**
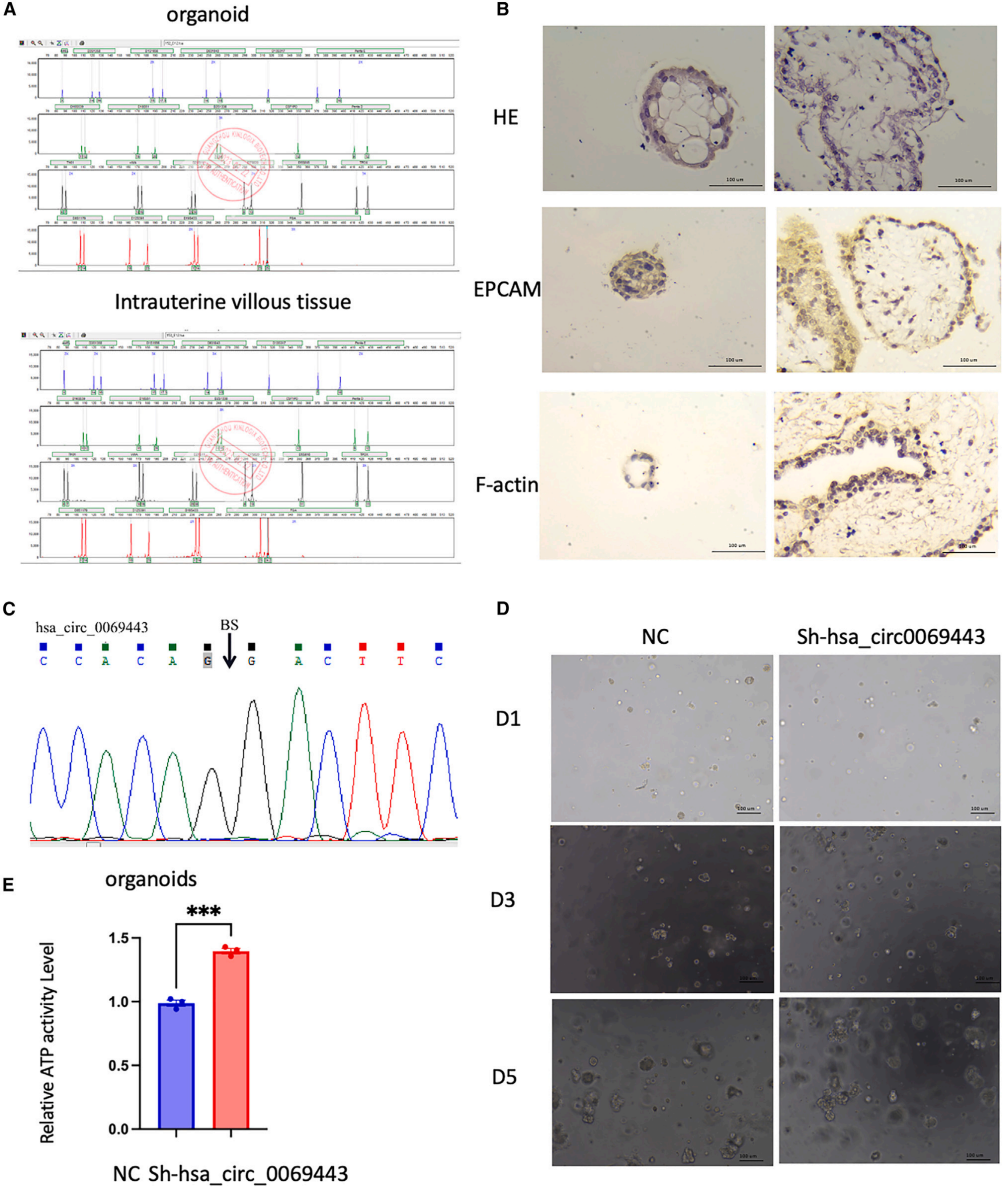
hsa_circ_0069443 promotes the proliferation of trophoblast like organs (A and B) STR identification and HE staining confirmed the consistency between organoid and original tissues. Images were captured at a magnification of 200×. (C) Sanger sequencing confirmed the presence of hsa_circ_0069443 exists in organoids. (D and E) the hsa_circ_0069443 overexpression group showing increased organoid activity compared with that of the control group. Images were captured at a magnification of 40×. Scale bar = 100 μm in (B, D). The results shown are representative of three separate experiments, *n* = 3. Data are expressed as the mean ± SEM. ∗∗∗, *p* < 0.001. SEM, standard error of the mean.

To find a better *in vivo* research model, we constructed a trophoblastic organoid model, and confirmed the consistency between organoid and original tissues through simple tandem repeat (STR) identification and hematoxylin and eosin (HE) staining (Figures 8A and 8B). Sanger sequencing verified that hsa_circ_0069443 was expressed in organoid tissues (Figure 8C). The hsa_circ_0069443 overexpression Lentivirus was transfected into organ-like cells, overexpression of Lentivirus stably transfected organ like cells, and their growth and activity of were observed. On the fifth day of observation, obvious differences could be seen, with the hsa_circ_0069443 overexpression group showing increased organoid activity compared with that of the control group (Figures 8D and 8E), further indicating that hsa_circ_0069443 promotes the proliferation of trophoblast cells.

## Discussion

In this study, we discovered that hsa_circ_0069443 is an important circRNA that is downregulated in patients with early pregnancy loss and thus might directly cause early pregnancy loss. Pregnancy is a complex and inefficient process, and the key to successful pregnancy lies in the implantation of embryos and the development of the placenta.^25^ In the physiological process of early placental pregnancy, CTBs differentiate into STBs and EVTs. STBs mainly participate in processes such as material exchange and hormone secretion between the mother and the fetus. EVTs migrate and invade into decidual tissue and one-third of the myometrium, fix the placenta to the uterine wall, and participate in the regulation of the maternal-fetal interface immune tolerance and the recasting of uterine spiral arteries.^26^^,^^27^ The migration and invasion of EVTs in the decidua and myometrium comprise a series of very important processes from embryo implantation to development. Disturbance of any step in this process might lead to pregnancy loss. Multiple studies have found that excessive proliferation and invasion of trophoblasts might be related to hydatidiform mole, choriocarcinoma, or placental implantation, while insufficient proliferation and invasion are associated with pregnancy loss.^28^^,^^29^

Does the downregulation of hsa_circ_0069443 lead to insufficient proliferation and invasion of trophoblasts, leading to loss of early pregnancy? First, we confirmed that knockdown of hsa_circ_0069443 in HTR-8/SVneo cells, JEG-3 cells, and explants inhibited cell proliferation, migration, and invasion. We further explored the molecular mechanism of early pregnancy loss caused by hsa_circ_0069443. Currently, there are three known main mechanisms by which circRNAs exert their biological functions. First, circRNAs in the nucleus can regulate gene expression at the transcription and splicing levels.^30^ The second is that circRNAs can be translated and act via an encoded protein.^31^ The third widely proven feature is that circRNA can act as a sponge for miRNAs through miRNA binding sites to regulate miRNA activity toward other target genes.^32^^,^^33^^,^^34^ Proteins are the direct effectors of life activities; however, research on the interaction between circRNAs and proteins is limited. Some studies have shown that circRNAs can serve as protein bait, scaffolds, and recruiters.^35^^,^^36^^,^^37^ Herein, we aimed to explore whether circRNAs bind to RBPs to exert their role. We searched using biological information websites (http://www.csbio.sjtu.edu.cn/bioinf/RBPsuite/) and confirmed through western blotting, RIP, and RNA pulldown experiments that hsa_circ_0069443 combined with ALKBH5. Previous reports have shown that knocking down *ALKBH5* promotes trophoblast invasion in HTR-8/SVneo cell lines and villous explants, while overexpression of *ALKBH5* inhibits cell invasion. *In vitro* and *in vivo* experiments have shown that ALKBH5 plays an important role in trophoblast cell invasion.^21^ The present study further confirmed that ALKBH5 can promote the proliferation of trophoblasts. This study explored whether the role of hsa_circ_0069443 in miscarriage depends on its regulatory effect on ALKBH5. The results showed that while knocking down hsa_circ_0069443, the slowed cell proliferation, migration, and invasion caused by hsa_circ_0069443 knockdown could be restored by si-ALKBH5 transfection. Additionally, the elevated ALKBH5 expression and reduced FN1 expression induced by hsa_circ_0069443 knockdown could also be restored. This indicates that the main mechanism of action of hsa_circ_0069443 in miscarriage is through the regulation of ALKBH5 protein expression.

ALKBH5 is an m6A demethylase belonging to the AlkB subfamily of the Fe (II)/2-oxoglutarate (2OG) dioxygenase superfamily. N6 methyl adenosine (m6A) is the most common internal modification in mammalian mRNA.^38^^,^^39^ M6A modification regulates mRNA stability, selective splicing, transportation, and translation,^40^^,^^41^^,^^42^^,^^43^ thereby affecting various biological processes, and some studies have shown that m6A modification is involved in implantation, placental formation, and pregnancy maintenance.^44^ Hsa_circ_0069443 is connected to ALKBH5; therefore, hsa_circ_0069443 might affect the overall level of m6a methylation in trophoblasts. Consequently, we verified that knockdown of hsa_circ_0069443 reduced the overall methylation level of trophoblast cells. Furthermore, in HTR-8/SVneo and JEG-3 cells, knockdown of hsa_circ_0069443 decreased the overall methylation level compared with that in the normal controls, while overexpressing hsa_circ_0069443 increased the overall methylation level, which could be restored after knocking out *ALKBH5*.

This study used RIP-seq, biological information network prediction, methylation site prediction, and searching for target mRNA. Finally, *FN1* was determined as the target of ALKBH5. The relationship between hsa_circ_0069443, ALKBH5, and *FN1* mRNA was verified. FN family member Fibronectin (FN1) is a high molecular weight glycoprotein. It is expressed in many cell types and has various biological functions, participating in cell adhesion, growth, migration, and differentiation.^45^^,^^46^ Previous studies have shown that FN1 is closely related to the proliferation, invasion, and adhesion of trophoblast cells.^47^^,^^48^ According to research by JI et al., FN1 is downregulated in the villous tissue of both patients with spontaneous abortion and in spontaneous abortion mice. *In vitro* and *in vivo* experiments have confirmed that FN1 is involved in cell proliferation and apoptosis. According to Kyoto Encyclopedia of Genes and Genomes (KEGG) and gene ontology (GO) enrichment analysis and western blotting validation, FN1 regulates trophoblast cell apoptosis through the phosphatidylinositol-4,5-bisphosphate 3-kinase (PI3K)/protein kinase B (AKT) signaling pathway, which is important for regulating cell proliferation and apoptosis. Knocking out *FN1* in mice induced apoptosis of normal mouse trophoblast cells. We further demonstrated through MeRIP and RNA stability analysis that has_circ_0069443 regulates the stability and expression of *FN1* in trophoblast cells through m6A methylation-dependent regulation, thereby participating in the occurrence of early pregnancy loss.

Successful pregnancy is an extremely complex process, and multiple studies have confirmed the role of exosomes in pregnancy. The mother-fetus communication mediated by exosomes is the key to embryo implantation. The interaction between the mother’s uterus and the embryo determines successful pregnancy, especially in the early pregnancy stage, during which most pregnancy losses occur.^48^^,^^49^^,^^50^^,^^51^^,^^52^ The external trophoblast cells are crucial for the connection between the embryo and the maternal uterus. EVTs can adhere to the uterine wall, migrate, and invade the uterus, ultimately forming the placenta.^53^ To study has_circ_0069443 in terms of the relationship between the fetus and the mother, we first clarified that the extracellular vesicles of trophoblasts were rich in hsa_circ_0069443. The exosomes were incubated with hESC, compared with the negative control group, the addition of exosomes promoted hESC cell migration and invasion. Multiple studies have shown that during the implantation process, the endometrium undergoes EMT, promoting embryo attachment and adhesion.^24^^,^^54^ This study also confirmed that co-incubation of adding exosomes that overexpressed hsa_ circ_ 0069443 and the endometrium can promote the EMT process. We confirmed that hsa_circ_0069443 participates in maternal-fetal interactions through the secretion of exosomes by trophoblasts.

To study trophoblast cells *in vivo*, selecting an appropriate animal model poses certain difficulties. Mammals vary greatly in the degree of invasion of trophoblast cells into the uterus, from non-invasive to invasive penetration through uterine blood vessels and direct contact with maternal blood.^55^ Mice are the most used animal models, similar to humans, where EVTs come into direct contact with maternal blood. However, unlike humans, the degree of decidualization and invasion of the uterus in mice varies.^56^ The invasion of the trophoblast in the placentas of apes is very similar to that of humans; however, ethical and economic considerations limit their use in research. In recent years, the development of 3D trophoblast organoids culture systems has provided a good model to study the function of human trophoblast organoids.^57^^,^^58^ Human trophoblast organoids grow in a complex 3D structure, which is very similar in biology and function to the villous placenta *in vivo*.^59^ Therefore, we overexpressed hsa_circ_0069443 from a lentivirus in cultured organoids, and compared with that in the control group, the trophoblast organoids like activity was enhanced, further clarifying that hsa_circ_0069443 simulates internal functions.

### Limitations of the study

Our research found that hsa_circ_0069443 binds to the demethylase ALKNH5 and thereby affects FN1 mRNA methylation levels, which regulates its mRNA stability. However, this article still lacks some validations of previous experiments. For example, it remains to be further verified whether FN1 regulates the apoptosis of trophoblast cells through the PI3K/Akt signaling pathway and whether the absence of FN1 at the maternal-fetal interface hinders the intravascular construction of trophoblast cells and decidual vascular formation.

## Resource availability

### Lead contact

Further information and requests for resources and reagents should be directed to and will be fulfilled by the lead contact, Xiujie Sheng (2008691150@gzhmu.edu.cn).

### Materials availability

This study did not generate new unique reagents.

### Data and code availability

• RNA-seq data have been deposited at SRA(SRA: PRJNA1170415) and is publicly available as of the date of publication. All data presented in this study will be shared upon reasonable request by the lead contact, Dr. Xiujie Sheng (2008691150@gzhmu.edu.cn).
• This paper does not report original code.
• Any additional information required to reanalyze the data reported in this paper is available from the lead contact upon request.

## Acknowledgments

We thank the Guangdong Provincial Medical Science and Technology Research Fund project (Project No.: A2023337) for providing financial support for this research. We thank The Third Affiliated Hospital of Guangzhou Medical University, Guangzhou, China for providing the research platform and convenient experimental conditions.

## Author contributions

X.-J.S. and Y.Z. designed the research; B.-X.L. and M.-Y.W. conducted the experiments, analyzed the data, and wrote the paper; D.-M.Z., J.-Q.L., and Z.-H.W. contributed to the specimen collection; B.-F.L. and X.-L.L. repeated verification of the experimental data. All authors read and approved the final manuscript.

## Declaration of interests

All authors declare no competing interests.

## STAR★Methods

### Key resources table

**Table:** 

REAGENT or RESOURCE	SOURCE	IDENTIFIER
**Antibodies**		

Rabbit polyclonal anti-ALKBH5	Proteintech	Cat#:16837-1-AP; RRID:AB_2242665
Rabbit polyclonal anti-FN1	Proteintech	Cat#:15613-1-AP; RRID:AB_2105691
Rabbit polyclonal anti-glyceraldehyde-3-phosphate dehydrogenase (GAPDH)	Bioworld	Cat#:AP0063; RRID:AB_2651132
Rabbit polyclonal anti-Tubulin	Bioworld	Cat#:AP0064; RRID:AB_2797447
Rabbit polyclonal anti-CD63	Proteintech	Cat#:25682-1-AP; RRID:AB_2783831
Rabbit polyclonal anti-TSG101	Proteintech	Cat#:28283-1-AP; RRID:AB_2881104
Rabbit polyclonal anti-HSP70	Proteintech	Cat#:10995-1-AP; RRID:AB_2264230
Rabbit polyclonal anti-E-Cadherin	Immunoway	Cat#:YT1454; RRID:AB_3073633
Rabbit polyclonal anti-MMP-2	Immunoway	Cat#:YT2798; RRID:AB_2814759
Horseradish peroxidase (HRP)-conjugated goat anti rabbit lgG secondary antibody	Bioworld	Cat#:BS13278; RRID:AB_2773728
Rabbit IgG	Proteintech	Cat#:B900610; RRID: AB_3674206
Anti-N6-methyladenosine (m6A)	MilliporeSigma	Cat#:ABE572-I; RRID:AB_2892214
M6A Monoclonal antibody	Proteintech	Cat#:68055-1-lg; RRID:AB_2918796
CD49f Antibody	Miltenyi Biotec	Cat#:130-119-807; RRID:AB_2751859
HLA-G antibody [MEM-G/9] (PE)	GeneTex	Cat#:GTX78335; RRID:AB_625690
Anti-Human CD32	Stemcell	Cat#:18520; RRID:AB_2925215

**Biological samples**		

Chorionic tissues	This paper	N/A

**Chemicals, peptides, and recombinant proteins**		

FBS	Excell Bio	FSP100
Extracellular serum	VivaCell Biotechnology	C3801-0100
1% penicillin/streptomycin antibiotics	Procell Life	PB180120
1% sodium pyruvate	Procell Life	PB180422
0.25%Trypsin-EDTA	NCM Biotech	C100C1
MEM	Procell Life	PM150410
RPMI-1640	Procell Life	PM150110
DMEM/F-12(1:1) basic(1X)	Gibco	8122199
opti-MEM medium	Thermo Fischer	31985062
PBS	Procell Life	PB180327
Matrigel	BD Biosciences	356234
Matrigel	Corning	356234
Mitomycin C	Selleck	S8146
NP40	Beyotime	P0013F
PMSF	Beyotime	ST506
Protein A/G magnetic beads	Bimake	B23202
Actinomycin D	Selleck	S8964
Proteinase K	Beyotime	ST535-100mg
RNase A	Solarbio	R8021
Trizol	Accurate Biotechnology	AG1102
Hifair® Ⅲ 1st Strand cDNA Synthesis SuperMix for qPCR (gDNA digester plus)	Yeasen	11141ES
Hieff® qPCR SYBR Green Master Mix(Low Rox Plus)	Yeasen	11202ES
RIPA	Bestbio	BB-32011
Immobilon-P PVDF membrane 0.45 μm	MilliporeSigma	IPVH00010
Amersham Hybond-N+ chambers	Pall	66485
Fast blocking buffer	NCM Biotech	P30500
Red blood cell lysis buffer	Solarbio	R1010
CellCounting Lite 3D	Vazyme	DD1102
TrypLE™Express	Gibco	12604021
Pancreatin	Woheng Bio	N/A
Trophoblast organoids culture medium	Woheng Bio	N/A

**Critical commercial assays**		

DNFU Tissue Dissociation Kit	GEXSCOPE	11300602
EasySep™ PE Positive Selection Kit II	Stemcell	17684
EpiQuik m6A RNA Methylation Quantitative Kit	EpiGentek	P-9005
ECL chemiluminescence kit	Yeasen	36208
RNA pull-down kit	BersinBio	Bes5102
Exosomes extraction kit	Yeasen	41201ES25
Cell Counting Kit 8 (CCK-8) assay	Dojindo	TT796
PAGE Gel Fast Preparation Kit	EpiZyme	PG111-PG113
BCA Protein Quantification Kit	Takara Bio	T9300A
Coomassie blue Kit	Beyotime	P0017A

**Deposited data**		

RNA-seq	This paper	PRJNA1170415
RIP-seq	This paper	Table S6

**Experimental models: Cell lines**		

JEG-3	Procell Life	CL-0127
HTR-8/SVneo	Procell Life	CL-0765
hESC	Professor Zhao Yang’s research group	N/A
EECs	Professor Zhao Yang’s research group	N/A

**Oligonucleotides**		

Primers for qPCR, see Table S2	This paper	N/A
Si-NC or EV	RiboBio	N/A
OE-/SI-hsa_circ_0069443	RiboBio	N/A
SI-ALKNH5	RiboBio	N/A
SI-FN1	RiboBio	N/A
Biotin-labeled hsa_circ_0069443 RNA probes	BersinBio	QR379

**Software and algorithms**		

Adobe Photoshop	Adobe	https://www.adobe.com/es/
Graphpad Prism9.0	GraphPad Software	https://www.graphpad.com
ImageJ	ImageJ software	https://imagej.net/software/fiji/
EndNote X9	EndNote X9 Software	https://endnote.com/downloads
SPSS 26.0	SPSS software	https://www.ibm.com/spss

### Experimental model and study participant details

#### Patient characteristics

From January 2021 to May 2024, a total of 309 women of childbearing age who underwent induced abortion in the Third Affiliated Hospital of Guangzhou Medical University for Obstetrics and Gynecology were included in the study. Among them, 134 patients had aborted embryos confirmed by ultrasound, and those with the following characteristics were excluded: 1) Ultrasonography showed that the uterus had dysplasia; 2) Chromosomal microarray analysis detection of villi after miscarriage indicated chromosomal abnormalities; 3) Having infectious diseases; 4) Suffering from thyroid disease. Another 175 cases were women with normal pregnancy, who were included as healthy controls, and ultrasound confirmed the presence of fetal heartbeat during induced abortion. All recruited patients underwent artificial abortion and terminated pregnancy at 6–12 weeks. We have supplemented the clinical characteristics of these pregnant women and included the additional information in Table S5. We compared the age, gestational age, and BMI of these pregnant women using SPSS software and found no significant differences between the early pregnancy group and the early pregnancy loss group (Figure 1E). Chorionic and decidual tissues were collected from these patients, and washed twice with ice-cold physiological saline to remove surface blood. Some tissue samples were placed in tissue preservation solution for culture of explants and organoid experiments, while the remaining samples were placed in liquid nitrogen for subsequent experimental analysis. All tissue samples used for this study were obtained with written informed consent from all participants. The study was approved by the Medical Ethics Committee of the Third Affiliated Hospital of Guangzhou Medical University, Medical Research (No. 2022227).

#### Cell culture

JEG-3 cells, derived from human choriocarcinoma, exhibit similarities to primary cytotrophoblast cells and are commonly used as a research model for cytotrophoblasts.^60^ HTR-8/SVneo cells are commonly used as a model to study extravillous trophoblast (EVT) migration and invasion.^61^ The above cells were purchased from Wuhan Pricella Biotechnology Co.,Ltd. (Wuhan, China). The HTR-8/SVneo cell line was cultured in Roswell Park Memorial Institute (RPMI)1640 + 1% penicillin/streptomycin antibiotics (P/S; PB180120; Procell Life, Hyderabad, India) containing 10% fetal bovine serum (FBS; FSP100; Excel Bio, Shanghai, China). The JEG-3 cell line was cultured in minimal essential medium (MEM; PM150410; Procell Life) complete culture medium with 10% FB+1% P/S + 1% sodium pyruvate (PB180422; Procell Life). Normal endometrial stromal cells (hESC) and endometrial epithelial cells(EECs), a gift from Professor Zhao Yang’s research group, were cultured in 10% FBS +1% P/S MEM complete culture medium. The cell incubator was set at a constant temperature of 37° C, with a CO_2_ saturation level of 5%. All cell lines were validated by STR profiling and tested negative for mycoplasma.

#### Isolated and cultured primary trophoblast cells

Specific method for CTB cell isolation and culture: Early pregnancy chorionic villi collected from the abortion room were placed on a 35mm culture dish on ice. Using a blade, the villous tissue was gently scraped, discarding the villous membrane. The villous tissue was then minced and transferred to a 15mL centrifuge tube. PBS was used to wash away the remaining villi at the bottom of the dish, which were also transferred to the centrifuge tube. The tube was centrifuged at 4°C, 500g, for 5 min, and the supernatant PBS was discarded. The villous tissue was suspended in digestion solution (DNFU Tissue Dissociation Kit, 11300602) and transferred to a 15mL centrifuge tube. The tube was sealed with a membrane and incubated in a 37°C water bath for 30 min. To terminate the digestion, 20% fetal bovine serum (FBS; FSP100; Excell Bio, Shanghai, China) supplemented DMEM/F12++ (1% penicillin-streptomycin, 10% FBS) was added. The mixture was filtered through a 1000μm cell strainer to remove debris. The unfil tered tissue was transferred to a new 15mL centrifuge tube, and 5-10mL of digestion solution was added according to the tissue quantity. The tube was again sealed with a membrane and incubated at 37°C for 5–10 min. After digestion was completed, the cells were washed and filtered through a 100μm cell strainer. The supernatant was discarded by centrifugation at 500*g*, 4°C for 5 min. The pellet was resuspended in 4 mL of 1X red blood cell lysis buffer, placed on ice for 5 min, and then terminated by adding 2% FBS PBS in a 1:1 ratio. After cell counting, the pellet was collected by centrifugation at 500*g*, 4°C for 5 min and resuspended in 0.1–2.5mL of culture medium (100μL). The cells were then transferred to a 5mL flow tube and mixed with 10μL of anti-Human CD32(Fc gamma Rll) Blocker (100μL/mL,18520,Stemcell, Canada), followed by the addition of CD49f antibody (5μL/10ˆ6 cells). After incubating at room temperature for 15 min, 10μL of Selection Cocktail (100μL/m, EasySep PE Positive Selection Kit II,17684,Stemcell, Canada) was added and incubated at room temperature for another 15 min. The mixture was vortexed for 30 s to ensure thorough dispersal. Subsequently, 5μL of RapidSpheres (50μL/mL, EasySep PE Positive Selection Kit II,17684,Stemcell, Canada) were added to the mixture, and after thorough mixing, it was incubated at room temperature for 5 min. Next, 2mL of 2% FBS PBS was added and thoroughly mixed by pipetting. The tube (without the cover) was placed onto a magnet and incubated at room temperature for 10 min. The tube was lifted from the magnet, and with continuous motions, the magnet and tube were flipped to discard the supernatant. This step was repeated three times. The cells were resuspended in 2% FBS DMEM, counted, and plated onto a 35mm culture dish.

For the establishment of primary EVT models: Please refer to the experimental methods for detailed descriptions of the extravillous explant culture. After anchoring the villous tissue for 2–3 days, non-adherent tissue fragments were removed, and the explants were further cultured for 8–12 days with medium changes every 2 days. Subsequently, the EVT cells were isolated from the extravillous explants using trypsin digestion for subsequent experiments. We have supplemented the clinical characteristics of these pregnant women in Table S3.

#### Explant culture

We obtained 2–3 mm small tissue sections from the top of human placental villi in early pregnancy (6–12 weeks), gently clamped the villi with tweezers and laid them flat in a 12 well culture dish pre-coated with Matrigel (BD Biosciences, Franklin Lakes, NJ, USA). Approximately 100 μL of Dulbecco’s modified Eagle’s medium (DMEM)/F12 medium containing 10% FBS was added to each well for cultivation. After approximately 24 h of anchoring, 100 nM of si-circ0069443 or si-NC were added to the wells according to the different groups, or 200 nM circ0069443 overexpression plasmid or empty vector control. The configuration of the transfection complex was carried out according to the steps in the Lipofectamine3000 Invitrogen, Thermo Fisher Scientific, Waltham, MA, USA) transfection reagent manual, and were similar to those used for cell transfection. The germination and migration of EVTs from the distal end of the villi were recorded daily, for up to 3 days. Image Pro Plus 6.0 software (Media Cybernetics, Rockville, MD, USA) was used to measure the degree of migration. All explant experiments conducted with cultured villi were repeated three times. We have supplemented the clinical characteristics of these pregnant women in Table S4.

#### Trophoblast organoids culture

Fresh villous tissue samples were collected from the placenta, immediately placed in tissue protection solution, stored on ice, and transported to the laboratory. The villus tissue was transferred to a 50 mL centrifuge tube containing DMEM/F12 + 1% P/S, 10 mM HEPES and 2mM GlutaMAX at room temperature, and washed in a shaker at low speed for 15 min. Then. 5–10 mL of pancreatin preheated at 37°C was added according to the amount of tissue (Woheng Bio, Guangzhou, China), and incubated under a sealed film in a 37°C water bath for 3–5 min. Then, 20% FBS (FSP100; Excell Bio, Shanghai, China) DMEM/F12 + 1% penicillin streptomycin, 10 mM HEPES, and 2 mM GlutaMAX was added to terminate digestion, and the sample was filtered through sterile gauze. The tissue remaining on the gauze was collected, transferred to a new 50 mL centrifuge tube, and the digestion was repeated After stopping digestion, the solution was pipetted up and down 10 times using a 1 mL pipette to further digest the tissue, which was filtered through a 100 μm cell filter screen. The supernatant was removed by centrifugation at 400 × *g* at room temperature for 5 min, and 10 mL DMEM/F12 + 1% penicillin streptomycin, 10 mM HEPES, and 2mM GlutaMAX was added for resuspension and the sample was transferred to a 15 mL centrifuge tube for repeated centrifugation. The supernatant was discarded. According to the amount of the pellet, trophoblast organoids culture medium (Woheng Biological, Guangzhou, China) was added for resuspension, and the pre thawed Matrigel (Corning Inc.) was mixed with cell suspension at a 1:1. A 24-well plate that had been preincubated for 2 h at 37°C was removed from the incubator, added with 50 μL of the mixed solution to each well, placed in the 37°C incubator for 5 min, and then inverted and solidified for 20 min. After solidification, 500μL of trophoblast organoids culture medium was added per well and the plate was placed in a cell incubator for cultivation. Growth of the trophoblast organoids was observed every day and the culture medium was replaced every 2–3 days.

### Method details

#### RNA sequencing and RIP-seq sequencing

RNA high-throughput sequencing was used to analyze the placental villus tissue samples of four groups of patients with early pregnancy loss and patients with a healthy early pregnancy. First, the raw data was filtered to remove adapter sequences and low-quality reads, resulting in high-quality data (clean data). Then, the clean data was aligned with the ribosomal RNA database to remove ribosomal RNA sequences, resulting in effective reads. The effective reads were mapped to the reference genome for circular RNA identification. Circular RNA identification was performed using CIRI/circexplore2. High-confidence circular RNAs were obtained from the analysis. Microarray hybridization and data collection were conducted by Ruibo Biology (Guangzhou, China).

We used RIP-seq to analyze the differences between ALKBH5 protein and IgG group LongRNA in JEG-3 cell lines, which was conducted by Epigenetic (Guangzhou, China). After quality inspection using a Bioptic Qsep100 Bio-Fragment Analyzer (Bioptic, New Taipei City, Taiwan), and sequenced on the Illumina high-throughput sequencing platform (Illumina Inc., San Diego, CA, USA) generating 150 bp paired end reads (PE150).

#### RNA extraction and reverse transcription

According to the manufacturer’s instructions, the TRIZOL reagent (RNAex Pro Reagent, Accurate Biotechnology, Hunan, China) was used to separate total RNA from tissues or cells. All specimens had OD260/OD280 values ranging from 1.8 to 2.0. Then, 5μg of RNA was reverse transcribed to cDNA using Hifair III 1st Strand cDNA Synthesis SuperMix for qPCR gDNA Digester Plus (Yeasen Biotech, Shanghai, China) comprising the RT-PCR step of the qRT-PCR protocol.

#### Quantitative real time fluorescence PCR (qPCR)

The qPCR step of the qRT-PCR procedure was performed using a Quantstudio3 real-time fluorescent quantitative PCR instrument (Thermo Fisher Scientific) using qPCR Green master mix (Yeasen Biotechnology). Statistical analysis of the qPCR results was conducted using the Ct value comparison method. Using the2-ΔΔ Ct method^62^ calculates the relative RNA expression and normalizes it to the internal reference gene, human U6 mRNA. The primers are listed in the supplementary materials (Table S2).

#### Western blotting

Total protein was extracted using Radioimmunoprecipitation assay (RIPA) protein lysis buffer (Bestbio, Shanghai, China) and quantified using a bicinchoninic acid (BCA) assay (Takara Bio, Shiga, Japan). A 7.5–10% Super PAGE Bis Tris gel (EpiZyme, Cambridge, MA, USA) was used to separate the protein samples (30 μg each). After electrophoresis, the proteins were transferred to a immobilon-P PVDF membrane 0.45um (MilliporeSigma, Burlington, MA, USA). The membrane was blocked in fast blocking buffer (NCM Biotech, Suzhou, China) for 10 min, and then incubated overnight with the primary antibodies at 4° C. The primary antibodies used were as follows: rabbit polyclonal anti-ALKBH5 (1:2000; 16837-1-AP; Proteintech, Wuhan, China), rabbit polyclonal anti-FN1 (1:2000; 15613-1-AP; Proteintech), rabbit polyclonal anti-glyceraldehyde-3-phosphate dehydrogenase (GAPDH)(1:20000; AP0063, Bioworld, Irving, TX, USA), rabbit polyclonal anti-Tubulin (1:30000; AP0064, Bioworld), rabbit polyclonal anti-CD63 (1:500; 25682-1-AP; Proteintech), rabbit polyclonal anti-TSG101 (1:10000; 28283-1-AP; Proteintech), rabbit polyclonal anti-HSP70 (1:10000; 10995-1-AP; Proteintech), rabbit polyclonal anti-E-Cadherin (1:1000; YT1454; Immunoway, Plano, TX, USA), and rabbit polyclonal anti-MMP-2 (1:1000; YT2798; Immunoway).Then, the membrane was incubated with horseradish peroxidase (HRP)-conjugated goat anti rabbit lgG secondary antibody (AP0063; 1:5000; Bioworld) at room temperature for 2 h. Then, the A and B solutions in the ECL chemiluminescence kit were mixed in a 1:1 ratio, and used to cover the membrane for imaging (ChampChemi 610, Sage Creation, Beijing, China).

#### Cell transfection

Transfection was achieved using Lipofectamine 3000. Hsa_circ_0069443, *ALKBH5*, and *FN1* knockdown were performed using small interfering RNA (siRNA). The plasmids overexpressing hsa_circ_0069443 (pCE-RB-Mam-EGFP has_circ_0069443) and empty vector (pCE-RB-Mam-EGFP empty) were purchased from Ruibo Biotechnology (Guangzhou, China). At 48 to 72 h after transfection, total RNA or protein were extracted for subsequent experiments.

#### CCK-8 detection of cell proliferation

To evaluate cell proliferation a Cell Counting Kit 8 (CCK-8) assay (Dojindo, Kumamoto, Japan) was used. The treated cells (3000 cells/well) were inoculated into a 96-well plate. The CCK-8 solution was added to each well at 0, 24, 48, and 72 h or 0, 12, 24, 48, and 60 h. The optical density (OD) value at 450 nm was then measured using an enzyme-linked immunosorbent assay (ELX808, Agilent BioTek, Santa Clara, CA, USA). We recorded and calculated the average absorbance of three to four wells for each group.

#### Transwell migration and invasion experiments

The Transwell cell migration assay and invasion system were used to measure cell migration and invasion *in vitro*. For the cell migration assay, after transfection, HTR-8/SVneo cells (60000 cells/well) were suspended in 200 μL of serum-free medium and placed in each upper Transwell chamber (8 μM pore size; Falcon, Corning Inc., Corning, NY, USA). Then, 600 μL of medium supplemented with 20% FBS was placed in the lower chamber. After incubation for 48 h, a cotton swab was used to remove cells adhering to the surface of the insert. The cells on the bottom of the chamber were fixed with 4% formaldehyde and stained with 1% crystal violet solution. At 100× magnification, three different fields of view under an optical microscope were randomly selected and the number of cells in each image was calculated. Similar to the migration experiment, in the invasion experiment, the filter membrane of each insert was pre coated with Matrigel (BD Biosciences; Matrigel: serum-free medium 1:8), incubated in a 37°C degree incubator for 4 h before use in the experiment.

#### Wound-closure (migration) assays

Transfected JEG 、HTR-8/SVneo or hESC cells were seeded evenly into a 6-well plate and allowed to reach near confluence. The cells were treated with 1 μg/mL mitomycin C (selleck, USA) for 1 h. A sterile 200 μL pipette tip was used to create a scratch in the cell monolayer. The floating cells were washed away with PBS. HTR-8/SVneo cells were supplemented with 1640 medium, while JEG and hESC cells were supplemented with MEM medium. The plate was incubated in a 37°C cell culture incubator for 48 h. Images of the scratch at the same position were captured, and the area of scratch closure was analyzed using ImageJ software as ((0h scratch area - 48h scratch area)/0h scratch area).

#### Immunoprecipitation

Immunoprecipitation (IP) lysis buffer (NP40, Beyotime, Jiangsu, China) and 1% phenylmethylsulfonyl fluoride (PMSF; ST506, Beyotime) were used to lyse the cells. After centrifugation at 12000RPM for 20 min, the lysate was mixed with anti-ALKBH5 antibodies (16837-1-AP; 4 μg), with protein or immunoglobulin G (IgG; B900610; 4 μg, Proteintech) used as the control group, and then mixed with protein A/G magnetic beads (B23202; Bimake, Houston, TX, USA; 40 μL/tube) at 4°C. The magnetic beads were washed using with IP washing buffer three times. TRIzol was then added for routine RNA extraction, followed by cDNA reverse transcription and qPCR to verify the relative levels of target RNA between the input group, the IgG group, and the ALKBH5 group.

#### MeRIP qPCR

To quantify the level of m6A modified *FN1* mRNA, methylated RNA IP (MeRIP) was performed. Total RNA was separated from treated HTR-8/SVneo cells. Then, 4 μg of anti-m6A antibody (ABE572-I; MilliporeSigma) and protein A/G magnetic beads (B23202; Bimake) containing PMSF and RNase inhibitors in IP buffer (NP40; Beyotime) were added and incubated overnight at 4°C. RNA (100 μg) was incubated with m6A antibodies in each buffer. RNA was extracted from the magnetic beads and reverse transcribed into cDNA for further qPCR. The primer sequence for *FN1* for MeRIP-qRT-PCR is shown in (Table S2).

#### Determination of N6 methyladenosine content

An EpiQuik m6A RNA Methylation Quantitative Kit (P-9005, EpiGentek, Farmingdale, NY, USA) was used to estimate the N-methyladenosine (m6A) content of RNA according to the manufacturer’s instructions.PC(provided by the reagent kit) was used to prepare six different concentration standard curves. Total RNA was extracted from HTR-8/SVneo cell samples under different treatments. RNA samples with OD260/OD280 > 1.9 and OD260/OD230 > 1.7 were selected. Subsequently, 200 ng RNA from different HTR-8/SVneo cell samples and negative controls from each group (provided by the kit) were added to specific tubes (provided by the kit). An enzyme-linked immunosorbent assay (ELX808, Agilent BioTek) was applied and the OD value at 450 nm was used to quantitatively analyze the content of m6A in each sample.

#### DOT blot experiment

Trizol was used to extract total RNA, and 1μg and 5 μg of total RNA at 75°C for 5 min, immediately placed on ice to cool for 1 min, and then 1μg and 5μg of RNA were dropped onto Amersham Hybond-N+ chambers (0.22m, 66485,Pall,USA). After drying, the membrane was crosslinked twice using ultraviolet cross linker. blocked using 5% skim milk for 30 min, and incubated overnight with anti-m6A antibodies (1:1000; 68055-1-lg; Proteintech) at 4°C. The membrane was washed with Tris-buffered saline Tween 20 (TBST) three times, for 10 min each time, incubated with anti-mouse secondary antibodies at room temperature for 1 h, and washed three times using TBST. A and B solutions from the ECL chemiluminescence kit were mixed in a 1:1 ratio and incubated with the membrane for 5 min before imaging (ChampChemi 610).

#### RNA pulldown assay

The BersinBio RNA pull-down kit(BersinBio, Guangzhou, China), the BersinBio RNA pull-down kit was used according to the manufacturer’s protocol. In short, biotin-labeled RNA probes were coupled to magnetic beads and incubated with cell protein extracts. The protein molecules that specifically bound to the RNA probes were eluted to obtain the target RNA probe-protein complex. RNA or proteins were extracted for qRT-PCR or western Blotting analysis.

#### RNA stability test

HTR-8/SVneo cells were inoculated into each well of a 6-well plate, and after the cells adhered to the wall, the HTR-8/SVneo cells were transfected with si-circ0069443 and negative control (NC) oligonucleotides, respectively. At 36–48 h after transfection, HTR-8/SVneo cells were treated using 5 μg/mL actinomycin D (S8964; Selleck, Houston, TX, USA) and cell samples were collected at 0, 3, and 6 h, respectively. RNA was extracted for qRT-PCR analysis to detect the impact of different treatments on RNA stability.

#### Extracellular extracts preparation

Before collection, the cells were cultured in a medium containing 10% extracellular serum (VivaCell Biotechnology, Denzlingen, Germany) for at least 48 h. According to the manufacturer’s instructions, exosomes were extracted from the supernatant of HTR-8/SVneo cells using an exosomes extraction kit (YEASEN, 41201ES25). Proteins were extracted from exosomes and quantified using the BCA assay method. Exosomal biomarkers were detected using western blotting. The extraction of exosomes using ultracentrifugation, electron microscopy, and particle size detection were carried out by Beijing Hesheng Biotechnology Co., Ltd. (Beijing, China) according to standard protocols.

#### Overexpression of trophoblast organoids using lentiviral transduction

Carefully aspirate the organ culture medium from the wells, wash twice with PBS, and add 1 mL of organ passaging digestion solution (TrypLEExpress, Gibco, USA) to each well. Disperse the matrix gel by pipetting with a pipette gun (Corning, USA) and transfer to a 15 mL centrifuge tube. Place the tube in a shaking incubator at room temperature for 10–15 min. Add 10 mL of pre-chilled DMEM/F12+++ (1% penicillin-streptomycin, 10 mM HEPES, and 2 mM GlutaMAX) to resuspend the trophoblast organoids. Centrifuge at 300g for 5 min at 4°C, remove the supernatant, and add 10 mL of pre-chilled DMEM/F12 to resuspend the trophoblast organoids. Centrifuge at 300g for 5 min at 4°C, remove the supernatant, and add trophoblast organoids culture medium to resuspend the trophoblast organoids, adjusting the concentration to 10,000 per mL. Add lentivirus to the resuspension mixture at a volume ratio of 1:5 of lentivirus to resuspension mixture, and incubate in a 37°C incubator for 1–2 h. Centrifuge at 300g for 5 min at 4°C, remove the supernatant, and add an appropriate volume of trophoblast organoids culture medium and matrix gel in a 1:1 ratio. Take out the 96-well plate that was pre-incubated in a 37°C incubator 2 h in advance, add 5 μL of the mixture to each well, and invert the plate to allow the mixture to solidify for 20 min. After solidification, add 100 μL of trophoblast organoids culture medium to the 96-well plate and place it in a cell culture incubator for cultivation. Observe the growth and fluorescence of trophoblast organoids daily, and change the culture medium every 2–3 days. ATP activity should be measured after 7 days.

#### Trophoblast organoids activity detection

According to the instructions of the CellCounting Lite 3D (Vazyme, Nanjing, China) reagent, 100 μL of CellCounting Lite 3D (Vazyme) was added to 100 μL of the cell culture to be tested, shaken vigorously for 5 min to fully lyse the organoids, and placed at room temperature for 25 min to stabilize the luminescence signal. Luminescent was detected using a full wavelength enzyme-linked immunosensor.

### Quantification and statistical analysis

Use ImageJ software to measure the area of scratch closure, the degree of explant cell migration and the cell count. All data were analyzed using GraphPad Prism 9.0 and presented as the Standard Error of Mean (SEM) for a minimum of three independent experiments. The specific number of replicates for each experiment is indicated in the respective figure legend as number of n. All of the statistical details of experiments can be found in the figure legends and results. T-tests were employed for the comparison of two groups, and curve comparisons were conducted using two-way ANOVA. Statistical significance was considered as *p* < 0.05. ∗*p* < 0.05; ∗∗*p* < 0.01; ∗∗∗*p* < 0.001; ∗∗∗∗*p* < 0.0001; ns, not significant.

## Supplemental information

Document S1. Figure S1 and Tables S1–S4

Table S5. Patient Information of these pregnant women. (This excel form contains 2 sheets, ST1: Patient Information of normal early pregnancy), related to Figure 1

Table S6. RNA immunoprecipitation sequencing (RIP-seq) was performed by pulling down ALKBH5 in the JEG-3 cell line, related to STAR Methods
