# Supplementary material for: Mechanism of hsa_circ_0069443 promoting early pregnancy loss through ALKBH5/FN1 axis in trophoblast cells
Source: iScience. 2024 Dec 16;28(1):111608. doi: 10.1016/j.isci.2024.111608 (PMC11758834; doi:10.1016/j.isci.2024.111608)
Supplement: Document S1. Figure S1 and Tables S1–S4 [file mmc1.pdf]

iScience, Volume *28*

Supplemental information

Mechanism of hsa_circ_0069443 promoting early pregnancy loss through ALKBH5/FN1 axis in trophoblast cells

Bai-xue Li, Mei-yao Wu, Zhi-hui Wang, Dong-mei Zhou, Jian-qi Li, Bing-feng Lu, Xiao-ling Lin, Yang Zhao, and Xiu-jie Sheng

Table S1. The potential site of m6A-modified FN1 according to SRAMP

#	Position	Sequence context	Structural context	Local structure visualization	Score(binary)	Score(knn)	Score(spectrum)	Score(combined)	Decision
1	552	GAGUA AACCU GAAGC<br>UGAAG AGACU UGCUU<br>UGACA AGUAC ACUGG	N/A	N/A	0.740	0.731	0.586	0.678	m^6A site (Very high confidence)
2	616	GACAC UUAUG AGCGU<br>CCUAA AGACU CCAUG<br>AUCUG GGACU GUACC	N/A	N/A	0.638	0.586	0.68	0.652	m^6A site (High confidence)
3	631	CCUAA AGACU CCAUG<br>AUCUG GGACU GUACC<br>UGCAU CGGGG CUGGG	N/A	N/A	0.726	0.808	0.592	0.677	m^6A site (Very high confidence)
4	744	CACCU GGAGG AGACC<br>ACAUG AGACU GGUGG<br>UUACA UGUUA GAGUG	N/A	N/A	0.637	0.489	0.523	0.584	m^6A site (Moderate confidence)
5	843	GUGUU UUGAU CAUGC<br>UGCUG GGACU UCCUA<br>UGUGG UCGGA GAAAC	N/A	N/A	0.69	0.654	0.425	0.582	m^6A site (Low confidence)
6	978	AUGCA ACGAU CAGGA<br>CACAA GGACA UCCUA<br>UAGAA UUGGA GACAC	N/A	N/A	0.638	0.691	0.439	0.561	m^6A site (Low confidence)
7	1314	CCAAG AGACA GCUGU<br>AACCC AGACU UACGG<br>UGGCA ACUCA AAUGG	N/A	N/A	0.602	0.666	0.592	0.601	m^6A site (Moderate confidence)
8	1412	CCACA GAAGG GCGAC<br>AGGAC GGACA UCUUU<br>GGUGC AGCAC AACUU	N/A	N/A	0.582	0.674	0.556	0.576	m^6A site (Low confidence)
9	1618	AAGUG GUGUG GGACC<br>ACACA GAACU AUGAU<br>GCCGA CCAGA AGUUU	N/A	N/A	0.686	0.572	0.493	0.603	m^6A site (Moderate confidence)
10	1785	UGGGA AUGGU CGUGG<br>GGAAU GGACA UGCAU<br>UGCCU ACUCG CAGCU	N/A	N/A	0.662	0.422	0.501	0.585	m^6A site (Moderate confidence)
11	1891	CAUGA AGAGG GGCAC<br>AUGCU GAACU GUACA<br>UGCUU CGGUC AGGGU	N/A	N/A	0.698	0.685	0.455	0.600	m^6A site (Moderate confidence)
12	1962	CGACC AAUGC CAGGA<br>UUCAG AGACU GGGAC<br>GUUUU AUCAA AUUGG	N/A	N/A	0.713	0.525	0.446	0.597	m^6A site (Moderate confidence)
13	3234	UGGGA GGGAG AGCAA<br>GCCUC UGACU GCUCA<br>ACAGA CAACC AAACU	N/A	N/A	0.546	0.55	0.650	0.588	m^6A site (Moderate confidence)
14	3318	UUCUA CUGUC CUGGU<br>GAGAU GGACU CCACC<br>UCGGG CCCAG AUAAC	N/A	N/A	0.683	0.726	0.623	0.661	m^6A site (High confidence)
15	3377	CCGUG GGCCU UACCC<br>GAAGA GGACA GCCCA<br>GGCAG UACAA UGUGG	N/A	N/A	0.551	0.366	0.607	0.564	m^6A site (Low confidence)
16	3561	UCCAC CUUAC AACAC<br>CGAGG UGACU GAGAC<br>CACCA UUGUG AUCAC	N/A	N/A	0.548	0.654	0.561	0.558	m^6A site (Low confidence)
17	3743	CCAUC CAAGU CCUGA<br>GAGAU GGACA GGAAA<br>GAGAU GCGCC AAUUG	N/A	N/A	0.692	0.482	0.499	0.604	m^6A site (Moderate confidence)
18	3802	ACACC AUUGU CUCCA<br>CCAAC AAACU UGCAU<br>CUGGA GGCAA ACCCU	N/A	N/A	0.561	0.500	0.631	0.585	m^6A site (Moderate confidence)
19	4216	CCUAU UUUUG AAGAU<br>UUUGU GGACU CCUCA<br>GUAGG AUACU ACACA	N/A	N/A	0.686	0.575	0.564	0.632	m^6A site (High confidence)
20	4264	ACAGG GCUGG AGCCG<br>GGCAU UGACU AUGAU<br>AUCAG CGUUA UCACU	N/A	N/A	0.618	0.742	0.607	0.620	m^6A site (High confidence)
21	4633	CUUGA UUCCC CAACU<br>GGCAU UGACU UUUCU<br>GAUAU UACUG CCAAC	N/A	N/A	0.652	0.59	0.676	0.659	m^6A site (High confidence)
22	5282	UAACC ACCAC UCCCA<br>AAAAU GGACC AGGAC<br>CAACA AAAAC UAAAA	N/A	N/A	0.632	0.57	0.553	0.597	m^6A site (Moderate confidence)
23	5331	AGGUC CAGAU CAAAC<br>AGAAA UGACU AUUGA<br>AGGCU UGCAG CCCAC	N/A	N/A	0.636	0.723	0.612	0.630	m^6A site (High confidence)
24	5612	GCUCA UCCGU GGUUG<br>UAUCA GGACU UAUGG<br>UGGCC ACCAA AUAUG	N/A	N/A	0.757	0.647	0.599	0.688	m^6A site (Very high confidence)
25	5659	GUGAG UGUCU AUGCU<br>CUUAA GGACA CUUUG<br>ACAAG CAGAC CAGCU	N/A	N/A	0.636	0.584	0.619	0.626	m^6A site (High confidence)
26	6292	AUCUC AUGGG CCCCA<br>UUCCA GGACA CUUCU<br>GAGUA CAUCA UUUCA	N/A	N/A	0.632	0.342	0.53	0.576	m^6A site (Low confidence)

Table S2.Primer and siRNA sequences.

Species	Gene	Primer	qPCR primer sequence (5'→3')
Human	hsa_circ_0069443	F	TGCACTTCTTACTTGGGTCCG
		R	CGGCATGATTAAGGGCTTGG
	ALKBH5	F	CGGCGAAGGCTACACTTACG
		R	CCACCAGCTTTTGGATCACCA
	FN1	F	CGGTGGCTGTCAGTCAAAG
		R	AAACCTCGGCTTCCTCCATAA
	FN1-m6A	F	CTAGGCAATGCGTTGGTTTG
		R	GCAAGTCTCTTCAGCTTCAGGTT
	U6	F	CTCGCTTCGGCAGCACA
		R	AACGCTTCACGAATTTGCGT
	GAPDH	F	CCCCTTCATTGACCTCAAC
		R	CAAAGTTGTCATGGATGACC

Name	sequence (5'→3')
has_circ0069443 siRNA	CACCACAGGACTTCCAATA
ALKBH5 siRNA	GATCGCCTGTCAGGAAACA
FN1 siRNA	GGAAAACACTATCAGATAA

Table S3. Patient Information of primary trophoblast cells.

Sample ID	Age	Race	Sex	Number of v	Gestational	Childbearin	Height	Weight	BMI	Conception	Categories of Abortion
1	34	Asian	Female	7	7	G4P2	1.63	75	28.22838647	Natural conception	normal early pregnancy
2	35	Asian	Female	8	8	G5P2	1.62	73	27.81588173	Natural conception	normal early pregnancy
3	29	Asian	Female	8+2	8+	G2P1	1.65	53	19.46740129	Natural conception	normal early pregnancy
4	36	Asian	Female	7+5	7+	G3P1	1.59	57	22.54657648	Natural conception	normal early pregnancy
5	25	Asian	Female	7+2	7	G1P0	1.56	59	24.24391847	Natural conception	normal early pregnancy
6	35	Asian	Female	7+1	7+	G4P3	1.52	59	25.5367036	Natural conception	normal early pregnancy
7	28	Asian	Female	8+2	8+	G2P1	1.57	61	24.74745426	Natural conception	normal early pregnancy
8	42	Asian	Female	7+3	7+	G3P2	1.64	59	21.93634741	Natural conception	normal early pregnancy
9	34	Asian	Female	7	7	G4P2	1.63	75	28.22838647	Natural conception	normal early pregnancy
10	36	Asian	Female	6+4	6+	G3P1	1.53	64	27.339912	Natural conception	normal early pregnancy
11	35	Asian	Female	8	8	G5P2	1.62	73	27.81588173	Natural conception	normal early pregnancy
12	29	Asian	Female	8+2	8+	G2P1	1.65	53	19.46740129	Natural conception	normal early pregnancy
13	36	Asian	Female	7+5	7+	G3P1	1.59	57	22.54657648	Natural conception	normal early pregnancy
14	25	Asian	Female	7+2	7	G1P0	1.56	59	24.24391847	Natural conception	normal early pregnancy
15	29	Asian	Female	7+6	7+	G4P1	1.55	49	20.39542144	Natural conception	normal early pregnancy
16	33	Asian	Female	7+2	6	G2P1	1.56	51.5	21.16206443	Natural conception	early pregnancy loss
17	27	Asian	Female	7+2	6+	G3P1	1.62	59	22.48132907	Natural conception	early pregnancy loss
18	34	Asian	Female	9	7	G2P1	1.58	54.5	21.83143727	Ovulation induction	early pregnancy loss
19	34	Asian	Female	8+4	7+	G1P0	1.54	57	24.03440715	Natural conception	early pregnancy loss
20	34	Asian	Female	7+3	6+	G5P1	1.68	66	23.38435374	Natural conception	early pregnancy loss
21	30	Asian	Female	8+1	6+	G3P0	1.55	57	23.72528616	Natural conception	early pregnancy loss
22	30	Asian	Female	8+4	7+	G2P1	1.53	57.5	24.56320219	Assisted reproductive	early pregnancy loss
23	41	Asian	Female	8+5	7+	G21P2	1.63	63	23.71184463	Natural conception	early pregnancy loss
24	37	Asian	Female	7+5	6+	G4P0	1.58	48	19.22768787	Natural conception	early pregnancy loss

Table S4. Patient Information of explant culture.

Sample ID	Age	Race	Sex	Number of v	Gestational	Childbearin	Height	Weight	BMI	Conception	Categories of Abortion
1	29	Asian	Female	8+5	8+	G2P1	1.5	53	23.55555556	Natural conception	normal early pregnancy
2	43	Asian	Female	7+6	7+	G3P2	1.54	56	23.61275089	Natural conception	normal early pregnancy
3	42	Asian	Female	7+4	7+	G3P1	1.59	56	22.15102251	Natural conception	normal early pregnancy
4	33	Asian	Female	8	8	G3P1	1.67	66	23.66524436	Natural conception	normal early pregnancy
5	26	Asian	Female	7+2	7+	G1P0	1.63	52.5	19.75987053	Natural conception	early pregnancy loss
6	24	Asian	Female	8+1	8+	G2P1	1.7	82.5	28.5467128	Natural conception	early pregnancy loss
7	28	Asian	Female	8+5	7+	G1P0	1.6	59.5	23.2421875	Natural conception	early pregnancy loss
8	34	Asian	Female	8	8	G6P3	1.65	72	26.44628099	Natural conception	normal early pregnancy
9	27	Asian	Female	8+3	8+	G2P1	1.64	58	21.56454491	Natural conception	normal early pregnancy
10	29	Asian	Female	7+6	7+	G4P1	1.55	49	20.39542144	Natural conception	normal early pregnancy
11	33	Asian	Female	6+6	6+	G2P1	1.58	60	24.03460984	Natural conception	normal early pregnancy
12	27	Asian	Female	7+6	7+	G4P2	1.67	55	19.72103697	Natural conception	normal early pregnancy
13	36	Asian	Female	9	7+	G3P1	1.58	61	24.43518667	Natural conception	early pregnancy loss
14	31	Asian	Female	8+2	7+	G1P0	1.62	60	22.86236854	Natural conception	early pregnancy loss

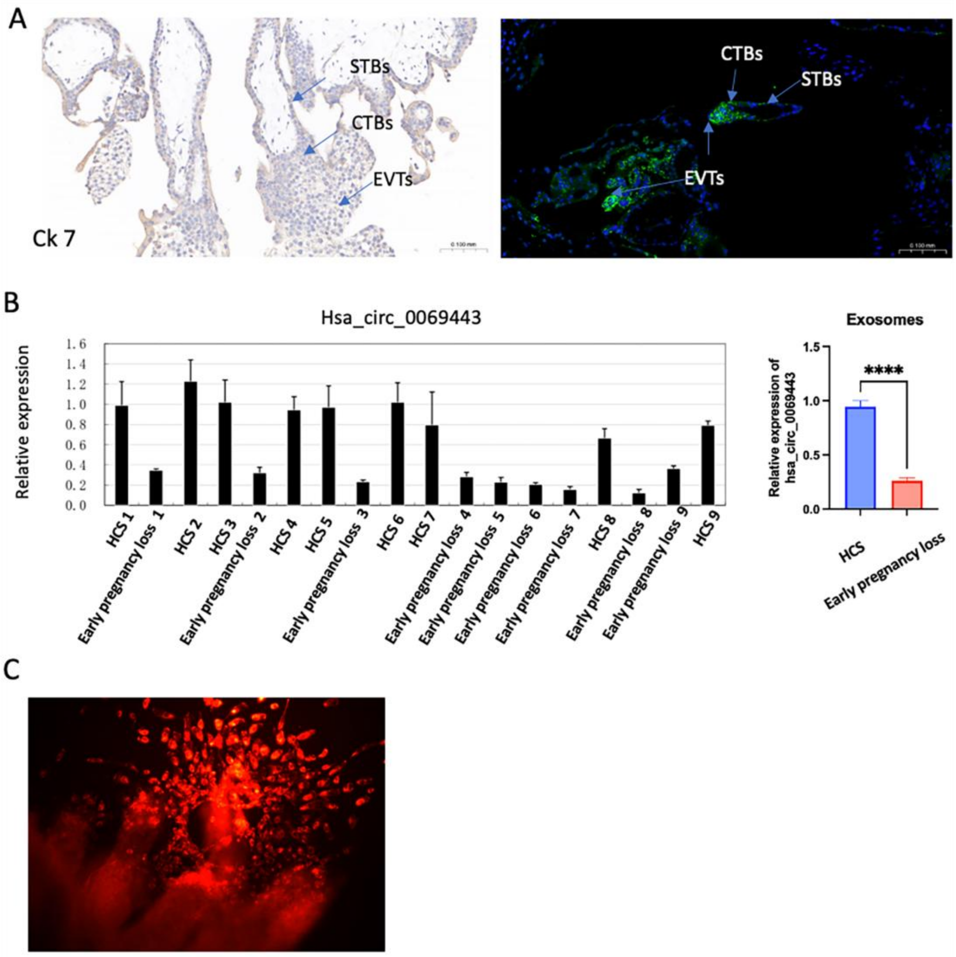

Figure S1. The expression of hsa_circ_0069443 in different cell types of human chorionic villi and its expression in exosomes isolated from abortive tissue or trophoblast.(A) CK7 staining and fluorescence in situ hybridization were performed on tissue sections, hsa_circ_0069443 is present in cytotrophoblasts (CTB), syncytiotrophoblasts (STB), and extravillous trophoblasts (EVT), with a greater abundance in CTB and EVT. n=5.(B) Isolated and cultured primary trophoblast cells from 9 pairs of early pregnancy loss and normal pregnancy chorionic villi tissues. Isolated exosomes from cell culture supernatants, followed by RNA extraction, and conducted qRT-PCR analysis. The results revealed a significantly decreased expression level of hsa_circ_0069443 in the exosomes derived from trophoblast cells of patients with early pregnancy loss. (C) Strong red fluorescence was observed at 24 hours after Cy3-siRNA transfection into villous explants. Data are expressed as the mean ± SEM. P ≥ 0.05; *, P < 0.05; **, P < 0.01; ***, P < 0.001; *****, P < 0.0001.
